# Exploring the Electrochemical Stability Window of an All-Solid-State Composite Cathode via a Novel *Operando* Tender XPS Setup

**DOI:** 10.1021/acsami.5c01672

**Published:** 2025-06-18

**Authors:** Rebecca Wilhelm, Robin Schuster, Tobias Kutsch, Simon Qian, Johannes Mahl, Tim Kratky, Johannes Wandt, Ethan J. Crumlin, Hubert A. Gasteiger

**Affiliations:** † 9184 Technical University of Munich, TUM School of Natural Sciences, Department of Chemistry and Catalysis Research Center, Chair of Technical Electrochemistry, Lichtenbergstraße 4, 85748 Garching, Germany; ‡ TUMint·Energy Research GmbH, Lichtenbergstraße 4, 85748 Garching, Germany; § Advanced Light Source, 1666 Lawrence Berkeley National Laboratory, Berkeley, California 94720, United States; ∥ Chemical Sciences Division, Lawrence Berkeley National Laboratory, Berkeley, California 94720, United States; ⊥ Technical University of Munich, TUM School of Natural Sciences, Department of Chemistry and Catalysis Research Center, Physical Chemistry with Focus on Catalysis, Lichtenbergstraße 4, 85748 Garching, Germany; # BMW AG, 80788 Munich, Germany; ∇ University of Agder, 4879 Grimstad, Norway

**Keywords:** all-solid-state batteries (ASSB), X-ray photoelectron spectroscopy (XPS), operando, Li_6_PS_5_Cl (LPSCl), argyrodite, Ni-rich NCM, Li-ion batteries, cathode electrolyte interphase (CEI)

## Abstract

All-solid-state batteries (ASSBs) have the potential to provide greater energy density than conventional batteries based on liquid electrolytes. Here, an *operando* ASSB cell setup for tender X-ray photoelectron spectroscopy (XPS) was developed, and the interface of a Ni-rich layered transition metal oxide cathode active material (CAM) and an Li_6_PS_5_Cl (LPSCl) solid electrolyte (SE) was evaluated during initial charge/discharge cycles. After validating the cell performance against a conventional pouch cell operated at high compression, intermittent galvanostatic cycling was performed, and XPS data were recorded as a function of state of charge (SOC). Upon the initial charge of the cell to ≈3.3 V_Li_, the LPSCl appears to decompose into LiCl, Li_3_PS_4_, and polysulfides, whose amount gradually increases with potential. Upon further charge, at a potential higher than *≈*3.8 V_Li_, initially, present sulfate and sulfite impurities decompose, and at ≈74% SOC (corresponding to a cathode potential of ≈4.10 V_Li_), surface reconstruction of the CAM particles due to lattice oxygen release is detected. In addition, at potentials beyond *≈*4.6 V_Li_, a decrease of the S 1s counts of the sum of the LPSCl, the thiophosphate, and polysulfide species suggests the formation of elemental sulfur that is lost via sublimation into the vacuum chamber.

## Graphical abstract

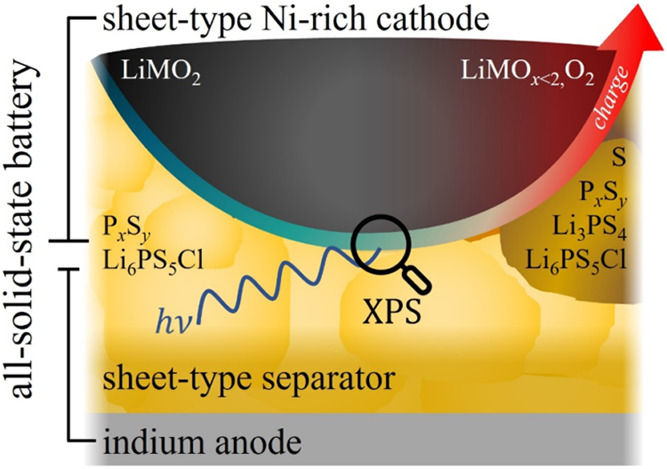

## Introduction

All-solid-state batteries (ASSBs) are a promising new battery technology, offering several possible advantages over liquid electrolyte-based lithium-ion batteries; however, their large-scale application has not yet been achieved.^1^ The investigated (quasi-)­solid electrolytes (SEs) are oxides,^2,3^ polymers,^4^ or sulfides,^5–7^ whereby for the latter the chlorine-substituted Li argyrodites Li_6_PS_5_Cl (LPSCl) are very promising.^8,9^ The high ionic conductivity (>3 mS cm^–1^ at room temperature) of LPSCl and related lithium thiophosphates allows for low polarization during cell operation,^10,11^ However, their main drawback is a narrow electrochemical stability window, so that they reportedly get oxidized to P_2_S_5_, S, and LiCl at potentials above ≈3.0 V vs Li^+^/Li (further on abbreviated as V_Li_).^12,13^ This potential is easily exceeded if layered transition metal oxides (LiMO_2_, M = Ni, Co, Mn) are used as cathode active material (CAM), as they are cycled to upper cutoff potentials of >4.0 V_Li_.^14^ Since this class of CAMs is necessary to achieve the energy densities required for most battery applications, it is crucial to understand not only the stability of the SE alone, but also the interaction of the SE and the CAM. For example, for an LPSCl-based ASSB cell, the decomposition of the SE after charge/discharge cycling was found to form a cathode/electrolyte interphase (CEI) around the CAM;^15^ later on, it was shown that the extent of SE decomposition can be reduced by coating the CAM particles with a protective layer.^16^ However, challenges arise when the CAM is cycled above ≈80% of its theoretical delithiation capacity, at which point the surface of layered transition metal CAM particles reconstructs upon the release of lattice oxygen, resulting in side reactions and impedance build-up.^14,17,18^

X-ray photoelectron spectroscopy (XPS) is a characterization technique that provides precise information regarding the elemental composition, oxidation states, and chemical environment.^19^ Furthermore, XPS is highly surface sensitive, and depending on the kinetic energy of the photoelectrons emitted from the core level of interest, the probing depth is only a few nanometers into the sample.^20^ In battery research, most XPS studies are conducted *ex situ*, using electrode samples harvested from battery cells, whereby the cell disassembly, the sample preparation, and/or the sample transfer can induce artifacts. Particularly in the case of ASSB electrodes, which are laminated to the separator and thus cannot be easily separated prior to the XPS measurements, often only fragments of the electrodes can be analyzed by *ex situ* XPS.^21^ These issues can be overcome by *operando* XPS measurements, as they would allow for the characterization of electrodes without cell disassembly. Freiberg and co-workers developed such an *operando* XPS cell setup by utilizing a propylene carbonate-soaked Li-ion conducting polymer; nonetheless, the operation of this setup at the partial pressure of the solvent led to severe evaporation, requiring the addition of a liquid reservoir to avoid electrolyte dry-out.^22^ For the study of ASSBs, the first *operando* XPS ASSB cell setup was reported by Wu et al.,^23^ composed of an (Li_2_S)_3_-P_2_S_5_ SE pellet sandwiched between an InLi anode and an LiCoO_2_/(Li_2_S)_3_-P_2_S_5_ composite cathode, whereby the latter was probed by the X-ray beam (Al K_α_ at 1486 eV); in this and in later work, the authors showed that the onset potential for the oxidative decomposition of (Li_2_S)_3_-P_2_S_5_ is at ≈2.7–2.9 V_Li_ and also suggested the formation of CoS_*x*_ species at ≈3.9 V_Li_.^24^ At about the same time, Davis et al.^25^ developed a setup to investigate Li plating and solid electrolyte interphase formation for two SEs, namely LPSCl and Li_10_GeP_2_S_12_. In a very recent study, Aktekin et al.^26^ examined the reductive decomposition of LPSCl by *operando* tender X-ray (4700 eV) XPS, for which they employed a 6 nm thick nickel film deposited on an LPSCl SE pellet as quasi photoelectron-transparent working electrode (WE) in combination with a lithium metal counter electrode (CE), showing the onset of LPSCl reduction to predominantly Li_2_S below ≈1.8 V_Li_. Ultimately, it would be desirable to conduct *operando* XPS measurements on ASSB cells prepared by state-of-the-art methods, i.e., with sheet-type SE separators and sheet-type composite cathodes based on Ni-rich CAMs.^11,27–30^ Furthermore, in the case of thiophosphate-based SEs like LPSCl, the use of tender X-rays allows to monitor the S 1s, P 1s, and Cl 1s signals with binding energies (BEs) ranging from 2140–2830 eV, whereby these singlet peaks enable an improved deconvolution of the S-, P-, and Cl-containing species compared to the S 2p, P 2p, and Cl 2p doublet peaks (from 125–205 eV) that are accessible with conventional lab X-ray sources. Therefore, the focus of this study is to develop an *operando* XPS ASSB cell setup that uses sheet-type composite electrodes and sheet-type separators, which then will be examined by *operando* tender X-ray XPS measurements.

The novel *operando* XPS cell setup developed in this study was applied for the investigation of the chemical and electrochemical interactions between an LPSCl SE and a nickel-rich CAM (LiNi_0.85_Co_0.10_Mn_0.05_O_2_) in a sheet-type composite cathode,^28^ which was subjected to charge/discharge cycles against an indium metal counter electrode. The cell was sequentially charged and discharged in two distinct voltage windows while tender XPS spectra were recorded during intermittent open circuit voltage (OCV) rest periods. The spectroscopic data were evaluated to get further insights into the oxidative stability limits of the composite cathode constituents.

## Experimental Section

### Materials

The handling of all materials and all processing steps was conducted inside an argon-filled glovebox (O_2_ < 0.1 ppm, H_2_O < 1 ppm, MBraun, Germany), unless described otherwise. Non moisture-sensitive materials and cell parts, stored under ambient atmospheric conditions, were dried at 70 °C under dynamic vacuum in a glass oven (Büchi B-585, Büchi, Switzerland) for at least 12 h prior to transfer into the glovebox. The LPSCl SE (*d*_50_ ≈ 5 μm for sheet-type separator, *d*_50_ ≈ 1 μm for composite cathode, Posco JK Solid Solution Co. Ltd., Korea), the carbon black super C65 (referred to as C65, TIMCAL, Switzerland), the indium foil (99.99% purity, 0.25 mm thickness, ChemPur, Germany), and the LiNi_0.85_Co_0.10_Mn_0.05_O_2_ (NCM, *d*_50_ ≈ 4 μm, BASF SE, Germany) CAM were used without further purification. The polyisobutylene rubber (PIB, Oppanol N150, average molecular weight *M*_w_ = 3.1 × 10^6^ g mol^–1^, BASF SE, Germany) was dried under dynamic vacuum at 70 °C for at least 24 h. Toluene (anhydrous, Merck, Germany) was dried over a molecular sieve (3 Å, Merck, Germany); its water content was determined to be <1 ppm by Karl–Fischer titration (Titro LineKF trace, Schott Instruments GmbH, Germany).

### Slurry Processing of the Separator and Cathodes

Sheet-type separators were prepared by combining 5.55 g of LPSCl powder and 0.45 g polyisobutylene rubber dissolved in a stock solution (3 wt % in toluene) in a flask (25 mL Duran Red GL, DWM Life Science, Germany), producing a slurry with a solids content of 30 wt %, which then was mixed with a speed mixer (ARE-250 CE, Thinky Corp., USA) for 10 min at 1000 rpm. The slurry was coated onto a siliconized polyester foil (PPI Adhesive Products GmbH, Germany) via the doctor blade technique, using a 1200 μm gap-bar (ERICHSEN, Germany). The deposited films were dried at 70 °C under dynamic vacuum for ≥12 h, yielding sheet-type separators with 7.5 wt % PIB and a dry-film thickness of ≈300 μm in its uncompressed state.

To prepare the sheet-type composite cathodes, NCM (87 wt %), LPSCl (10 wt %), C65 (2 wt %), and polyisobutylene rubber (1 wt %) were combined in a flask (25 mL Duran Red GL, DWM Life Science, Germany) with additional toluene to obtain a solids content of 30 wt % at a total solids content of 1 g. The slurry was mixed for 5 min at 2000 rpm and subsequently coated onto Al foil (16 μm, MTI, USA) with a 100 μm gap-bar. After drying at 70 °C under dynamic vacuum for ≥12 h, the resulting active material loading of the composite cathode was 1.1–2.0 mg_CAM_ cm^–2^ (≡0.3–0.5 mAh cm^–2^, referenced to the theoretical full delithiation capacity of the NCM of 275 mAh g_NCM_^–1^), whereby the exact CAM loading of each electrode could be determined with an estimated accuracy of roughly ± 10%. These low CAM loadings of the sheet-type cathodes were chosen to minimize the ionic resistance across the composite cathode, which resulted in a low cell polarization even at the low compression pressure in the *operando* cell (Figure 3).

### Cell Assembly

The electrode stack was prepared by a sequence of uniaxial compression steps using a hydraulic press (Atlas 15 T, Specac, U.K.). First, the sheet-type separator (16 mm diameter) was densified at a fabrication pressure of *p*_fab_ = 400 MPa. Subsequently, the sheet-type composite cathode (12 mm diameter) was laminated onto the precompressed separator at a lamination pressure of *p*_lam_ = 800 MPa for 2 min (Figure 1a). Finally, an indium metal CE (13 mm diameter, thickness ≈ 250 μm) was laminated onto the opposite side of the separator at *p*_lam_ = 100 MPa by a short pressing step of 5 s (Figure 1b), whereby the cell stack was sandwiched between two polypropylene disks to avoid short circuiting the cell via the pressing tool. Each of the thus prepared cell stacks was stored in a small glass vessel and prepared for transport by vacuum-sealing twice under an argon atmosphere. At the synchrotron facility, the samples were transferred into an argon-filled glovebox (O_2_ < 1 ppm, H_2_O < 1 ppm, MBraun, Germany) for cell assembly under inert conditions.

**Figure 1:**
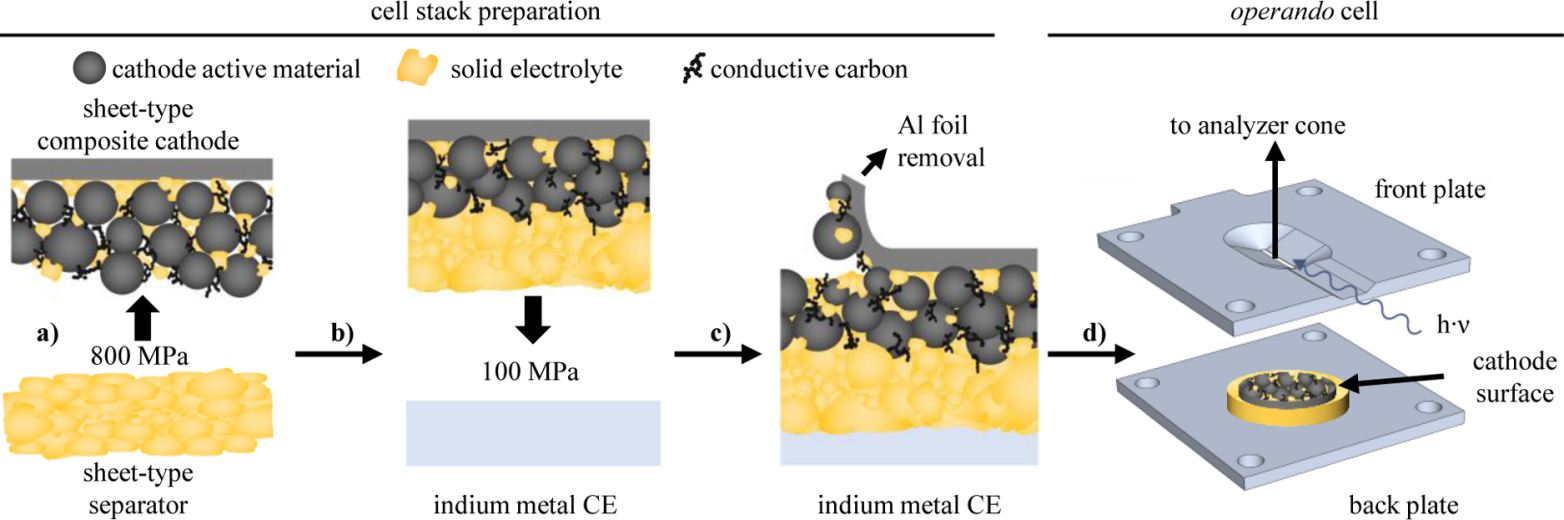
Schematic illustration of the individual assembly steps to prepare the cell stack and of the *operando* cell assembly. (a) Lamination of the sheet-type separator (precompressed at 400 MPa) onto the sheet-type composite cathode at a lamination pressure of 800 MPa. (b) Lamination of indium foil, serving as CE, onto the opposite side of the separator at 100 MPa. (c) Removal of the Al foil current collector from the composite cathode, whereby a minor part of the cathode constituents remains attached to the Al foil surface. (d) Schematic illustration of the cell stack placed onto the back plate of the *operando* cell, with the freshly exposed composite cathode facing toward the front plate; the back and front plates serve as current collectors for the CE and the cathode, respectively. Both plates are tightened by four insulated screws to apply a compression of ≈0.5–1 MPa onto the cell stack. XPS spectra from the composite cathode are acquired through the 1 mm × 10 mm slit in the center of the front plate.

During the third step, the aluminum foil onto which the composite cathode had been coated was removed (Figure 1c) to allow for the *operando* XPS analysis of the cathode. While the majority of the composite cathode was successfully transferred onto the sheet-type separator during the precompressing, a minor fraction of the composite cathode was found to stick to the Al foil. The mass of the remaining composite cathode on the cell stack was determined by subtracting the mass of the removed foil from the initially measured mass of the entire electrode, i.e., the composite cathode plus Al foil. This, however, leads to some uncertainty in the mass of the CAM remaining on the cell stack, so that the precise CAM mass was estimated from its known specific capacity (Figure S4). The electrode stack, with the composite cathode facing the analysis beam, was placed onto the stainless steel back plate of the *operando* XPS cell holder (Figure 1d) and then covered by the front plate that has a 1 mm × 10 mm slit through which the X-ray beam strikes the composite cathode surface; the slit was sufficiently large so that a new spot of the electrode could be analyzed for each SOC point, thereby avoiding any possible beam damage upon long-term exposure to the X-ray beam (Figure S5). Here it should be noted that owing to the high CAM content of the composite cathodes, the electrically nonconductive LPSCl SE and PIB binder fractions were rather small, resulting in a high in-plane electrical conductivity, so that a homogeneous charge/discharge of the composite cathode is expected also in the slit region that is not contacted by the front plate, which serves as current collector. This is evidenced by the absence of charging effects during XPS data acquisition.

The two plates were pressed together by four insulated screws that were tightened such that an effective operating pressure of *p*_oper_ ≈ 0.5–1 MPa is applied (determined by Prescale LLW, Fujifilm Corp., Japan); it must be noted, however, that there is no compressive force within the slit area. The plates constitute a part of the cell housing and serve as WE (front plate) and CE (back plate) current collectors that are connected to the Biologic potentiostat (SP-300, Biologic Science Instruments, France). The fully assembled cell was then attached to a 3D-printed cell brace (polylactic acid, Ender, Creality, China) in the glovebox, transferred to a glass flask (Schott, Germany), and mounted to the XPS end-station via an argon-filled glovebag under a continuous flow of argon (*t* < 5 min). A sheet-type separator was additionally mounted onto the front plate to monitor any possible exposure to moisture during the transfer into the end-station, which would result in the degradation of the LPSCl;^31,32^ the fact that no LPSCl degradation was observed in the XPS spectra (spectra at begin of test (BOT), Figure S15) validates the transfer procedure.

### Operando XPS

The *operando* XPS measurements were performed at beamline 9.3.1 of the Advanced Light Source (ALS) at the Lawrence Berkeley National Laboratory. The beam energy was set to 4000 eV, and the stability of the beam was tested on a control sample prior to the start of the experiment (Figure S5). The WE, i.e., the composite cathode, was grounded to the analyzer. The analysis chamber was then evacuated and reached a pressure of *p* < 5 × 10^–5^ Torr after ≈15 min and was maintained at *p ≈* 2 × 10^–6^ Torr until the end of the experiment. Prior to any electrochemical testing, beam-induced changes were ruled out by a separate cathode sample (Figure S5).

The cell was then charged in a first constant current (CC) mode step at a *C*-rate of *C*/10 (referenced to the full delithiation capacity and corresponding to a current of ≈30 μA) for 5 min, resulting in a charge corresponding to only ≈1% SOC to better resolve the initial increase in potential from BOT to “3.37 V_Li_”. While the cell was cycled vs an indium metal CE, the potentials given in this study are the cathode potentials referenced to V_Li_, since the potential of the indium CE reaches a stable value of +0.62 V vs Li^+^/Li after already a small amount of initial charge of the cathode,^33^ which in the present study was shown to be after an initial charge of ≈2% SOC (Figure S1). Potentials at which the CE was not yet stable are marked by adding quotation marks. This initial CC step was followed by a constant voltage (CV) phase for 30 min at the last measured potential (“3.37 V_Li_”) and an OCV phase of 45 min. All XPS spectra were acquired during the OCV phases, starting spectrum acquisition 5 min after setting the cell to OCV (Table S1); the duration of the OCV phase ranged between 60–120 min, depending on the number of XPS spectra that were acquired (see below). Only in a few instances, the OCV phase was extended to up to 150 min due to a loss of the X-ray beam. All subsequent charge/discharge steps were conducted in CC mode at *C*/10 for 60 min (ΔSOC ≈ 10%), each followed by a 30 min CV phase and an OCV phase. Using the above-described CC charge/discharge steps with intermittent CV-hold and OCV periods, the cell was charged in the first cycle until a cathode potential of 4.10 V_Li_ was reached and then discharged to a cathode potential of 2.50 V_Li_.

In the second cycle, the cell was charged up to a cathode potential of 5.50 V_Li_, using additional CV/OCV phases at 5.22 V_Li_ and 5.50 V_Li_ in order to increase the potential resolution at high SOCs. Please note that SOC is defined as the fraction of cycled delithiation capacity *x* in Li_1–*x*_MO_2_ with respect to the full theoretical delithiation capacity, i.e., 275 mAh g_NCM_^–1^.

During the OCV phases, the cell was first moved to a new spot on the sample, then the beam shutter was opened, and the focal point was calibrated, which took approximately 5–10 min. After that, spectra were recorded in the order of O 1s (6 scans, with ≈1 min/scan), Ni 2p (6 scans, with ≈2 min/scan), C 1s (3 scans, with ≈1 min/scan), S 1s (3 scans, with ≈2 min/scan) and S 2p (6 scans, with ≈2 min/scan); the calibration and spectral collection duration was ≈50 min. Prior to the first charge (i.e., at begin-of-test (BOT)), at the end of the first charge at 4.10 V_Li_, at the end of the first discharge at 2.50 V_Li_, and at the end of the second charge at 5.50 V_Li_, an additional survey scan as well as additional detail scans of the Co 2p, Mn 2p, Cl 1s, P 1s, P 2p, and Li 1s regions were recorded, resulting in a total measurement time of *≈*85 min in these instances (consequently, the OCV phases were increased to 120 min in these instances). After recording the spectra, the shutter was closed to minimize sample irradiation over the course of the experiment.

All spectra, unless stated otherwise, were energy aligned to the BE of the O 1s peak of the MO_2_ component of the NCM, which was fixed to 529.3 eV;^22,34,35^ throughout the entire experiment, the resulting BE shift correction remained essentially constant at 3.90 ± 0.03 eV and is ascribed to the work function of the electron energy analyzer. All counts per second (cps) at any given SOC were normalized to the area of the Ni 2p spectra that were acquired at the same SOC (Figures S7, S8). For spectral fitting, CasaXPS (version 2.3.24, Casa Software Ltd., U.K.) was used; the XPS peaks were fitted using a Gaussian–Lorentzian 30% blend fit function and a Shirley background was subtracted. To verify the observations, a second cell was assembled, charged to 5.50 V_Li_, and discharged to 2.50 V_Li_. This measurement showed full reproducibility of the first cell (data not shown). Errors for BEs and areas are given as the average of the absolute deviations to their mean value.

### Cross-Section Polishing

Cross sections were prepared by argon ion beam polishing, using a cross-section polisher of the type IB-19530CP (JEOL, Japan). A pristine battery cell stack was cut in half in an Ar-filled glovebox, fixed with adhesive copper tape (PPI Adhesive Products, Ireland) to the sample holder, and then inertly transferred to the device using an inert transfer shuttle (LB-11620TVCA, JEOL Japan) under an argon atmosphere. The sample was polished at −80 °C, first at 6 kV acceleration voltage for 4 h, followed by a second step at 4 kV for 2 h.

### Scanning Electron Microscopy (SEM)

SEM images were acquired using a JSM-IT200 InTouchScope (JEOL) field emission SEM instrument at 10 kV in secondary electron mode. The samples were prepared inside an argon-filled glovebox and transferred under an inert atmosphere to the SEM instrument. The samples were mounted quickly in the SEM chamber under air (<30 s), which led to an only minor degradation of the SE. To determine the elemental distribution, energy-dispersive X-ray spectroscopy (EDX) mappings were recorded at an incident electron beam voltage of 15 kV.

## Results and Discussion

### Microstructural Characterization of the Battery Cell Stack

Prior to electrochemical performance analysis of the *operando* cell, the microstructure of the cell stack was investigated by preparing a cross-sectional sample of the cell stack and analyzing it by SEM and EDX, which allows for a detailed analysis of the dimensions of the single cell stack layers and of the distribution of the LPSCl and the NCM particles. Figure 2a shows the cross-section of the whole cell stack: (i) the thin cathode layer on the top of the image (slightly darker in color, ≈5–8 μm in thickness), where the small, polycrystalline NCM particles are visible (magnified view in Figure 2b); (ii) the separator (with a thickness of ≈150 μm) in the middle part of the SEM image; and, (iii) the indium metal CE (with a thickness of ≈250 μm) of which a small part can be seen at the bottom part of the image. The magnified view in Figure 2b shows the boundary of the thin, sheet-type composite cathode and the sheet-type separator, illustrating that the cathode consists of only a few layers of secondary NCM particles and that the cathode appears to be homogeneous.

**Figure 2:**
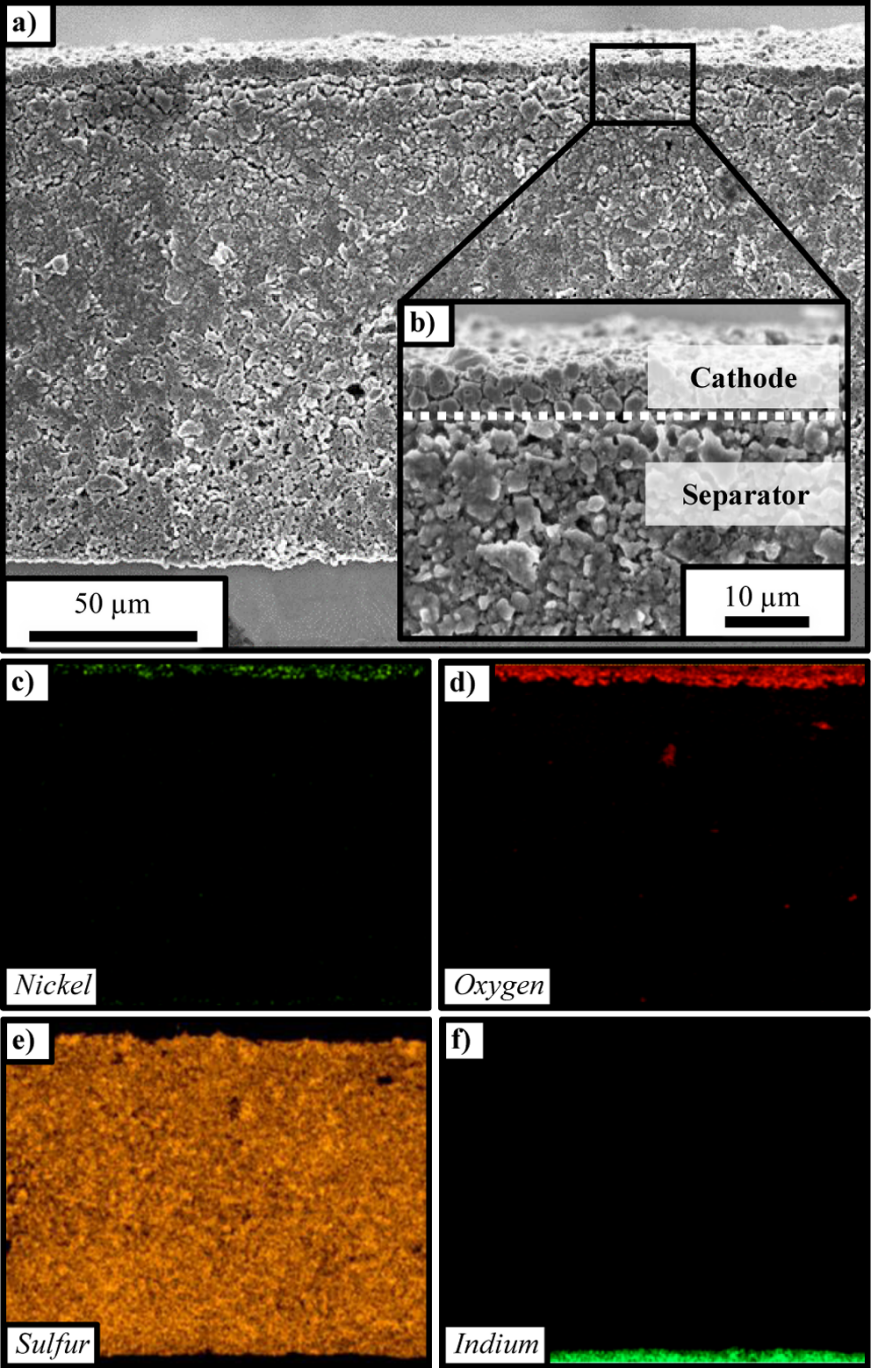
(a) Cross-sectional SEM images of the cell stack, with the thin, sheet-type composite cathode at the top, the sheet-type LPSCl separator in the middle, and a part of the indium CE at the bottom. (b) Zoomed-in SEM image of the cathode/separator interface. The middle/lower panels show EDX mapping images of a representative segment of the SEM image shown in panel a for: (c) nickel (yellow), (d) oxygen (red), (e) sulfur (orange), and (f) indium (green).

Figure 2c–f show EDX mapping images of a representative segment of the SEM image shown in Figure 2a. The EDX mapping further confirms the above assignment of the individual cell stack components: Figure 2c,d show the nickel (yellow) and oxygen (red) signals, respectively, which can be ascribed to the NCM in the composite cathode. By comparing these EDX maps to the SEM image in Figure 2a, it is evident that there are only 2–3 layers of CAM particles in the composite cathode, since the individual secondary NCM particles are clearly visible. The orange sulfur signal in Figure 2e depicts the sheet-type LPSCl separator, which is laminated to the indium CE on the bottom (Figure 2f, green). Overall, we can conclude that a thin and homogeneous cathode microstructure was obtained.

### Electrochemical Charge/Discharge of the Operando Cell

The validation of the electrochemical behavior of the *operando* cell is key to correlate each XPS spectrum to a certain SOC and cathode potential. With well-defined cell chemistry and a well-understood electrochemical response, the changes in the XPS features or the appearance of new features can then be correlated to specific onset potentials for the formation or decomposition reactions of the electrode components, which allows to determine the stability windows of certain chemical species.^36^ Thus, the degradation of the NCM material and the LPSCl SE at high potentials and the possible interrelationship can be monitored.^21,37^

The potential of the *operando* cell as a function of time is shown in Figure 3a, with a first charge to 4.10 V_Li_, a first discharge to 2.50 V_Li_, and a second charge to 5.50 V_Li_. Note that the potential corresponds to the cathode potential versus Li^+^/Li, except for the gray marked region in Figure 3a, where the potential of the indium CE is undefined, so that the cathode potential cannot be deduced from the cell potential (Figure S1). The charge (and discharge) procedure followed that of intermittent cycling and consisted of multiple sequences of *C*/10 CC charge (or discharge) steps, each followed by a CV-hold and an OCV period (this is illustrated for one sequence in Figure 3b). After the first charge sequence with a *C*/10 CC charge step for only 5 min, corresponding to a ΔSOC of ≈1%, a relatively large potential drop during the OCV phase was observed (≈0.3 V), which could be related to (i) a large charge transfer resistance (*R*_CT_) of the NCM at very low SOCs,^34^ (ii) an ongoing formation of a CEI upon reaching anodic the stability limit of the SE^38,39^ (Figure S3), and/or (iii) a variation of the CE potential that is poorly defined in this first charge sequence (Figure S1). In the subsequent charge sequences up to 4.10 V_Li_ in the first charge, the potential drop during the OCV phase was at most ≈70 mV, which is within the range of typical values for solid-state battery cells reported in the literature;^40^ during XPS data acquisition that started ≈5 min into the OCV phase, the change in OCV was at most ≈50 mV (Figure S2), resembling a reasonably well equilibrated state. During the first discharge to 2.50 V_Li_, the OCV potential drops were very similar and only increased once the NCM was fully lithiated again, which is likely related to the increased *R*_CT_ at low SOCs.^34^ During the second charge to 5.50 V_Li_, the OCV potential drop increased to ≈300–1200 mV at cathode CV-hold potentials of ≥4.60 V_Li_, which suggests that major parasitic reactions occur at these high potentials, promoting the continuous oxidation of the LPSCl SE. The significant oxidation of LPSCl at high potential was confirmed by measurements with an LPSCl/carbon composite model electrode (Figure S3). As the degradation of the LPSCl SE appears to be dependent mostly on potential (i.e., the higher the more pronounced), the CV-hold potential value (*U*_CV_) should correlate most closely with the LPSCl degradation processes, since during charge *U*_CV_ is always at least ≈30 mV higher than the subsequent OCV, whereby this difference increases drastically at very low or very high SOCs (Table S1).

**Figure 3:**
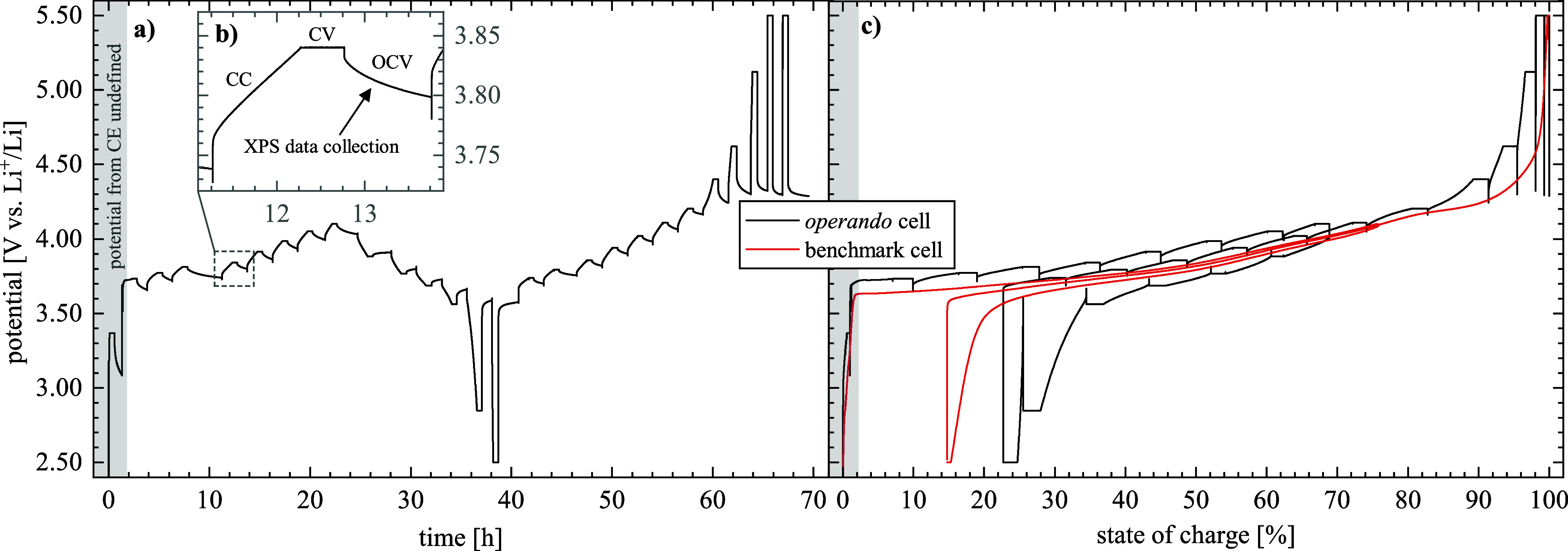
(a) Potential versus time profile obtained for the *operando* cell (black), with a first charge to 4.10 V_Li_, a first discharge to 2.50 V_Li_, and a second charge to 5.50 V_Li_. Except for the gray shaded area, in which the potential of the indium CE is poorly defined (Figure S1), the *y*-axis represents the cathode potential versus Li^+^/Li. The procedure was composed of a sequence of *C*/10 CC charge (or discharge) steps, with each being followed by a 0.5 h CV-hold and an OCV period during which XPS spectra were acquired; (b) magnification of a single sequence. The CC steps generally were conducted for 1 h (i.e., ΔSOC ≈ 10%), except for the first charge step that was only 5 min (i.e., ΔSOC ≈ 1%). (c) Potential versus SOC, comparing the *operando* cell (black) with a benchmark cell (red) that had a composite cathode from the same coating but was cycled at a lower rate of *C*/100 in a pouch cell under a compression of 30 MP. The gray shaded area applies for both cells and has the same meaning as stated above.

The potential versus SOC (i.e., the degree of delithiation) profile of the NCM cathode derived from the data in Figure 3a is shown in Figure 3c (again, the SOC region where the potential does not correspond to the cathode potential is marked by the gray highlighted area). Owing to the ≈10–20% error for the NCM loading in the *operando* cell (due to the very low loading and the removal of the aluminum foil, see Experimental Section), 100% SOC were defined to correspond to the charge capacity obtained after the second charge to 5.50 V_Li_ (Figure S4). The OCV potentials at the end of the OCV phase (*U*_OCV_, Table S1) at the different SOCs of the *operando* cell (black line) align nicely with the *C*/100 CC potential profile of a benchmark cell (red curve) that had the same cathode assembled in a pouch cell (compressed at 30 MPa; Figure S4). This closely applies for all SOC values, except at ≥95% SOC, which is reached for *U*_CV_ ≥ 4.62 V_Li_ where significant oxidation of the LPSCl SE apparently leads to a self-discharge reaction that is sufficiently large to strongly affect the OCV potential; however, except for these high-*U*_CV_ points, the *U*_OCV_ values of the *operando* cell closely correlate with the NCM SOC. Since the latter governs the onset of the surface reconstruction of the NCM particles due to lattice oxygen release (typically at ≈80% SOC), the *U*_OCV_ values are most representative when examining the formation of an O-depleted surface layer on the NCM particles (typically a rocksalt like layer for Ni-rich NCMs).^14,41^ In conclusion, despite the low compression of the *operando* cell (≈0.5–1.0 MPa), it exhibits reasonably low overpotentials and its potential versus SOC profile is in reasonably good agreement with that of a benchmark cell under the more conventionally used compression of 30 MPa.

### Analysis of the Operando O 1s Core Level Spectra

First, the spectroscopic data will be benchmarked in terms of the surface reconstruction of the NCM cathode active material, which is caused by the well-documented release of lattice oxygen at high SOC.^18,22,34,41^ The recorded O 1s spectra are shown in Figure 4 for the first charge to *U*_CV_ = 4.10 V_Li_ (Figure 4a), the first discharge to *U*_CV_ = 2.50 V_Li_ (Figure 4b), and the second charge to *U*_CV_ = 5.50 V_Li_ (Figure 4c), whereby the intensity is normalized to the total area of the Ni 2p spectrum recorded at the same SOC (Figure S7) in order to account for minor differences in the absolute intensity, which are most likely caused by moving to a new analysis spot on the cathode surface for XPS measurements after progressing to a new SOC. The respective SOC for each spectrum is indicated in the figure, following the color scale shown in the bottom-left corner of Figure 4; the correlation between the SOC and the associated values of the *U*_CV_ and the *U*_OCV_ (the potential at the end of the OCV phase) are listed in Table S1, and a more detailed analysis of the evolution of the Ni 2p spectra in *operando* is added to the Supporting Information (SI) in Figures S7 and S8.

**Figure 4:**
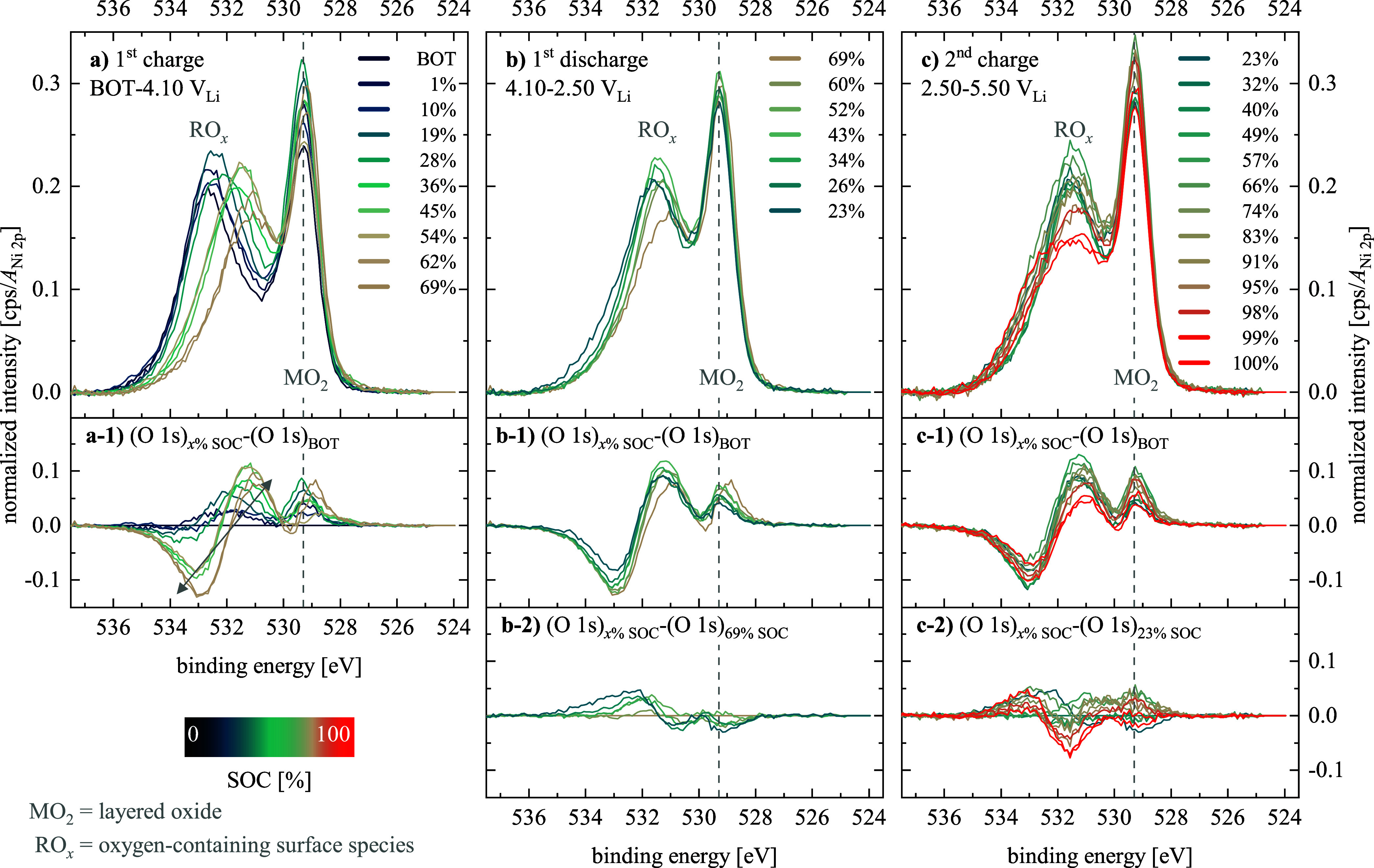
O 1s *operando* spectra (upper panels) obtained for (a) the first charge to *U*_CV_ = 4.10 V_Li_, (b) the first discharge to *U*_CV_ = 2.50 V_Li_, and (c) the second charge to *U*_CV_ = 5.50 V_Li_; the color coding (left bottom) marks the respective SOC. All spectra are normalized to the total Ni 2p peak area at the respective SOC (Figure S7). (a-1), (b-1), and (c-1): difference between the O 1s spectra at a given SOC (“*x* % SOC”) and the O 1s spectrum obtained at BOT. (b-2) and (c-2): difference between the O 1s spectra at a given SOC and the O 1s spectrum obtained at either the end of the first charge to 69% SOC (*U*_CV_ = 4.10 V_Li_, panel b-2) or at the end of the first discharge to 23% SOC (*U*_CV_ = 2.50 V_Li_, panel c-2). The *U*_CV_ and *U*_OCV_ values for each SOC are given in Table S1 of the SI. All spectra are BE-corrected to the O 1s feature from the lattice oxygen in the layered oxide structure of the NCM, here referred to as “MO_2_” (marked by the dashed vertical lines), which is fixed to be at 529.3 eV (Figure S11); furthermore, a Shirley background was subtracted from all spectra. The broad peak at higher BEs is ascribed to various oxygen-containing surface species and is referred to as “RO_*x*_”.

All O 1s spectra consist of two peaks, where the one at the lower BE corresponds to the oxygen in the layered phase of the NCM (further on referred to as “MO_2_”); as it is a relative narrow peak (full-width-at-half-maximum, FWHM ≈ 1.4 eV) with a well-defined peak position in each spectrum, its reported peak energy of 529.3 eV was used to reference the BE energy scale for all spectra (Figure S11).^22,34,35^ The O 1s feature at the higher BE (with a peak, e.g., at 532.5 eV at BOT) is relatively broad (FWHM ≈ 2.7 eV), suggesting the presence of multiple species. These are ascribed to both the commonly reported surface contaminants of nickel-rich NCMs such as Li_2_O, LiOH, and Li_2_CO_3_,^42–45^ all appearing within a narrow BE window from 529–533 eV,^44,46–48^ and to oxidative/chemical decomposition products of the LPSCl SE.^26,31,49^ Due to the absence of any additional distinguishing indicators, a deconvolution of the O 1s feature in this BE range (referred to as “RO_*x*_” feature, Figure 4) into distinct species is challenging. Thus, we plotted the O 1s spectra as *difference plots*, where Figure 4a-1, b-1, and c-1 show the difference of the O 1s spectrum at a given SOC (“*x*% SOC”) and that at BOT (i.e., as (O 1s)_*x*% SOC_–(O 1s)_BOT_); similarly, the bottom panels in Figure 4 show the difference of the O 1s spectrum at a given SOC and that taken at either the end of the first charge at 69% SOC (*U*_CV_ = 4.10 V_Li_, i.e., (O 1s)_*x*% SOC_–(O 1s)_69% SOC_ in Figure 4b-2) or at the end of the first discharge at 23% SOC (*U*_CV_ = 2.50 V_Li_, (O 1s)_*x*% SOC_–(O 1s)_23% SOC_ in Figure 4c-2).

During the first charge (Figure 4a), the MO_2_ feature centered about 529.3 eV retains a constant FWHM of 1.4 eV but slightly increases in intensity by ≈15%. This could be due to either an error introduced by the area normalization by the Ni 2p intensity or could indicate an attenuation effect; an overview of the used areas calculated for Ni 2p as a function of SOC are given in the SI, Table S2. Since the latter should be canceled out by the normalization to the area of the Ni 2p if it originated from the CAM, it might hint at a reaction unrelated to the active material. The offset of ≈3.1 eV between the RO_*x*_ and the MO_2_ peak stays constant until 10% SOC, after which the BE of the RO_*x*_ peak at ≈532.5 eV starts to shift toward lower BEs. After this initial BE shift of RO_*x*_, a nearly constant value of ≈531.4 ± 0.2 eV is obtained once an SOC of 36% (*U*_CV_ = 3.84 V_Li_) has been reached, decreasing the difference between the MO_2_ and the RO_*x*_ feature to ≈2.3 eV. Within the error of the measurement, the RO_*x*_ BE remains within this range also during the first discharge and during the second charge (Figure S11b). The origin of the RO_*x*_ BE shift can be examined by the respective difference spectra in Figure 4a-1, which exhibit an isosbestic point at ≈532 eV, suggesting that a species “*S*” transforms completely into a different species “S*”, as indicated by the gray arrow.^50,51^ As a first order estimate, the average area of the negative- and positive-going signals in the difference plot between ≈530–538 eV with respect to the area of the RO_*x*_ peak can be analyzed, and reveal that the overall area stays constant within the expected area, plateauing from *U*_CV_ > 3.84 V_Li_ (Table S3). While the BE range would fit well with the expected range for carbonates,^46,52^ they appear to degrade if *U*_CV_ > 3.8 V_Li_ (Figure S10), which contradicts the apparent constant area. Hence, the chemical nature of *S* and S* is still unclear and will be revisited later during the discussions of the sulfur species.

During the first discharge to 2.50 V_Li_ (Figure 4b), no significant changes in the O 1s spectra are visible, and the difference in BE between the MO_2_ and the RO_*x*_ feature remains constant, indicating that the shift of the RO_*x*_ BE during the first charge to 4.10 V_Li_ was not an electrostatic shift induced by the applied electrochemical potential but rather by an irreversible electrochemical reaction, namely the above suggested transformation of species *S* to S*. Hence, the difference plot relative to the BOT spectrum (Figure 4b-1) remains mostly unchanged, and the difference plots of the first-discharge O 1s spectra with respect to the spectrum at the end of the first charge to 4.10 V_Li_ (Figure 4b-2) only show a minor broadening of the RO_*x*_ feature toward higher BEs. The MO_2_ feature remains unchanged, with a consistently low FWHM of ≈1.3 eV. The BE would be close to the BE where Li_2_CO_3_ is usually detected, but is also close to where M-SO_*x*_ species are detected.^35,46,52,53^

During the second charge to 5.50 V_Li_ (Figure 4c), the RO_*x*_ feature begins to decrease in intensity at ≥83% SOC, i.e., for *U*_CV_ ≥ 4.21 V_Li_. At 5.50 V_Li_, the RO_*x*_ feature shows an increased FWHM of 3.4 eV at a BE of 531.5 eV. These changes are reflected by the decreased intensity of the difference plots of the O 1s spectra with respect to that at BOT (Figure 4c-1), which had remained completely unchanged during the first discharge (Figure 4b-1). This indicates that the S* species either reacted further or that any of the other oxygen-containing species reacted. Further insights can be obtained by constructing difference plots of the O 1s spectra with respect to that at the end of the first discharge (i.e., at 23% SOC or *U*_CV_ = 2.50 V_Li_), shown in Figure 4c-2: the negative peak at ≈531.7 eV suggests that oxygen-containing species at this BE are being consumed or transformed. On an absolute scale, the MO_2_ feature itself also seems to decrease slightly in intensity (Figure 4c), which can be seen more clearly in Figure 4c-1.

When comparing the spectral evolution of the MO_2_ feature in the difference plots referenced to the 2.50 V_Li_ spectrum (Figure 4c-2), the portion at lower BEs indicates the presence of side reactions, while the area is increasing at 529.8 eV, which is within the BE range of an O-depleted MO_*x*_ phase.^22,34,41^ This apparent oxygen depletion first appears at ≈74% SOC, i.e., after the CV phase at *U*_CV_ = 4.11 V_Li_. According to the literature, this SOC value is near the value that is reported for the formation of an O-depleted MO_*x*_ phase (≈80% SOC);^14,18,54,55^ the slightly lower SOC observed here might be related to the intermittent charging protocol used in the present study, with long CV-hold times for each SOC point.

### Analysis of the Operando Cl 1s, S 1s, and P 1s Core Level Spectra

In order to get further insights into the degradation of the LPSCl SE and the decomposition/transformation of surface contaminants over the course of the charge/discharge of the *operando* cell, the Cl 1s (blue), S 1s (green/brown/pink), and P 1s (red/orange) spectra (all measured at OCV) were examined at the different conditions shown in Figure 5: (a) at BOT; (b) after the first charge to *U*_CV_ = 4.10 V_Li_, corresponding to 69% SOC, (c) after the first discharge to *U*_CV_ = 2.50 V_Li_, corresponding to 23% SOC; and, (d) after the second charge to *U*_CV_ = 5.50 V_Li_, corresponding to 100% SOC. The associated profile of the cathode potential versus SOC is shown in the right-hand-side panel of Figure 5 (redrawn from Figure 3c).

**Figure 5:**
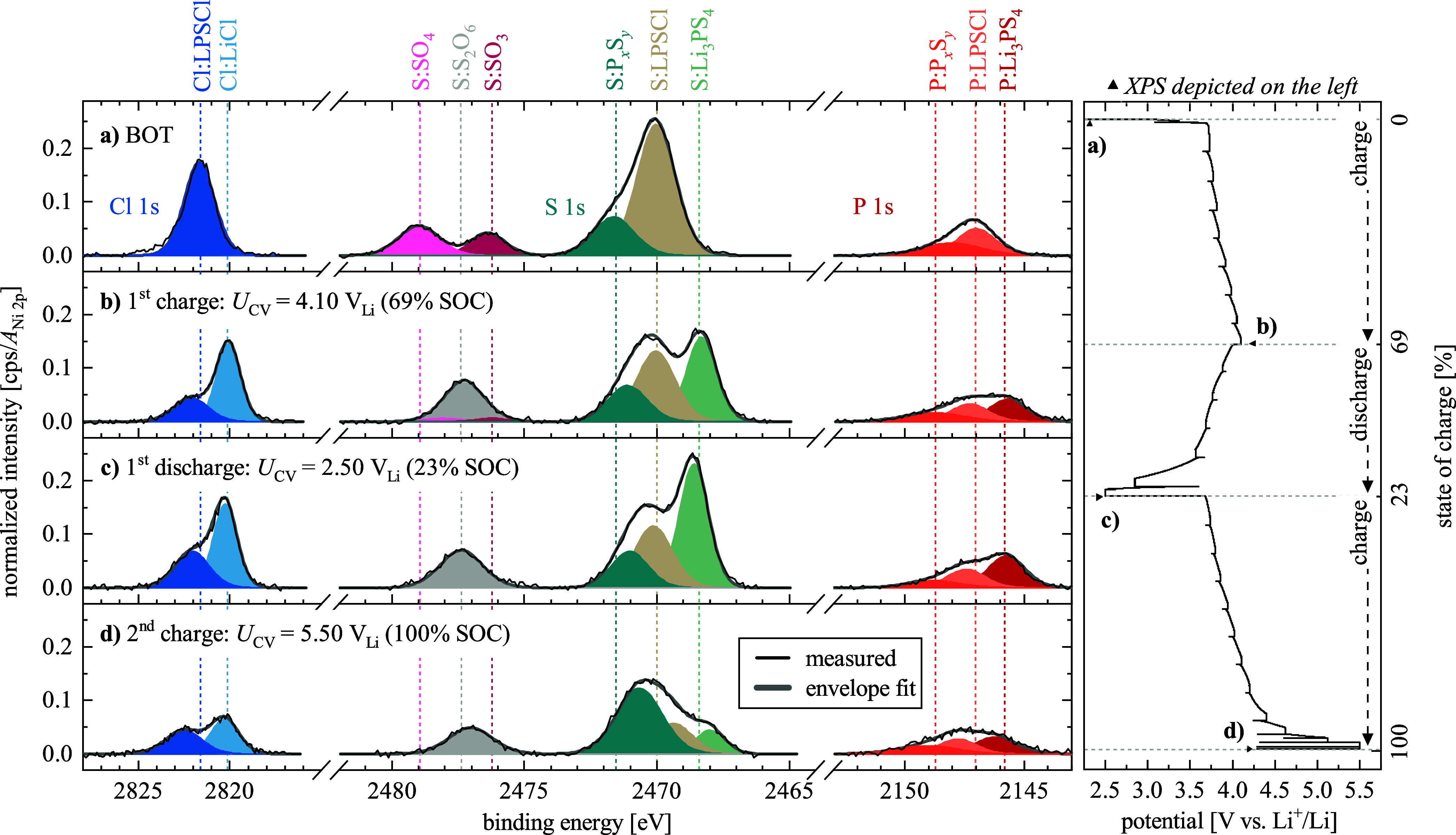
*Operando* XPS spectra of the Cl 1s, S 1s, and P 1s regions, acquired at different cell conditions: (a) at BOT; (b) after the first charge to *U*_CV_ = 4.10 V_Li_ (69% SOC); (c) after the first discharge to *U*_CV_ = 2.50 V_Li_ (23% SOC); (d) after the second charge to 5.50 V_Li_ (100% SOC). The right-hand-side panel shows the cathode potential versus SOC profile (redrawn from Figure 3c), marking the points where the here shown spectra were acquired. All spectra are normalized to the total Ni 2p peak area at the respective SOC and the BEs are referenced to the MO_2_ peak that was fixed to be at 529.3 eV (Figure S11). Detail fittings for the envelope fit (gray lines) were performed with a Gaussian–Lorentzian blend (30%) on top of the Shirley background, and the species associated with each component fit are indicated by the labels placed above panel a.

As a first step, the *operando* spectra acquired at BOT (Figure 5a) will be examined in order to assign the spectral features that correspond to the LPSCl SE. In the Cl 1s region, only one species is visible at 2821.7 eV (FWHM = 1.9 eV) and is ascribed to the chloride in the pristine LPSCl, which we will further on refer to as “Cl:LPSCl”.^26^ In the P 1s region, the main peak at 2147.1 eV (FWHM = 1.9 eV) can be ascribed to the phosphide species in the LPSCl (further on referred to as “P:LPSCl”).^26^ Finally, in the S 1s region, the main peak at 2470.0 eV (FWHM = 1.7 eV) can be ascribed to the sulfur in the species in the PS_4_^3–^ unit cell of pristine LPSCl (further on referred to as “S:LPSCl”).^26^ While the S 1s region of the sheet-type LPSCl separator (prepared by the same slurry-process and using the same LPSCl and PIB binder as for the sheet-type cathode) showed only the S:LPSCl species at 2470.0 eV (Figure S15b), additional S 1s features at ≈6 and ≈9 eV higher BE are observed for the sheet-type cathode in the *operando* measurements (see Figure 5a) and in analogous reference measurements (peaks labeled 1 and 2 in Figure S15a). Based on the linear increase of the S 1s BE with increasing sulfur oxidation state established by Yu et al. for different reference compounds,^56^ the sulfur species detected at 2476.4 eV (FWHM = 1.5 eV) would have an S^4+^ oxidation state, likely corresponding to a metal sulfite (further on referred to as “S:SO_3_”), and those detected at 2479.0 eV (FWHM = 1.8 eV) would have an S^6+^ oxidation state, likely corresponding to a metal sulfate (further on referred to as “S:SO_4_”). The BEs obtained for sulfite and sulfate reference samples and a comparison with measurements in the literature^26^ confirm these assignments (Table S4). Said species are also measured for the pristine NCM powder in the S 2p region, yet here a deconvolution into sulfate and sulfite is not straightforward (Figure S16c). Further, as said species were not present on the sheet-type separators (Figure S15b), their origin is unclear and may be explained by the following hypotheses:

(i) Sulfates could originate from the NCM synthesis process where Na_2_SO_4_ is formed as side product, which is known to migrate to the NCM particle surface upon the final calcination step.^57–59^

(ii) The formation of sulfites has been observed in literature before, where one suggested pathway is the reaction with oxygen.^37^ However, this seems unlikely, since no lattice oxygen release occurs for a fully lithiated NCM (i.e., at BOT) and oxygen leaking from ambient air would also imply the intrusion of water that would react with the LPSCl to Li_3_PO_4_ and LiCl,^31^ which are not observed. A chemical interaction of the thiophosphate SE with oxygen-containing surface functional groups of the conductive carbon would be another possible origin of the sulfites.^21,60^ Hence, a chemical interaction of the LPSCl with the other constituents of the composite cathode seems plausible and was also observed in a pristine cathode (Figures S15a and S16a). Based on the measurement of the S 2p region of the pristine NCM powder (Figure S16c), an unambiguous deconvolution into sulfates or sulfites is not possible, and the formation of sulfites upon calcination remains a possibility.

There is also a significant S 1s feature at 2471.6 eV (FWHM = 1.8 eV), i.e., very close to the S 1s feature of the pristine LPSCl. According to the study by Yu et al.,^56^ the BE of this feature would suggest an oxidation state between S^1–^ and S^0^. However, based on previous reports, this feature likely originates from polysulfides and species like P_2_S_7_^4–^ or P_2_S_6_^2–^;^16,23,26,37^ hence, it will further on be labeled as “S:P_*x*_S_*y*_”. To identify the phosphorus component of the S:P_*x*_S_*y*_ species, the P 1s spectrum is revisited, where also an additional feature is observed at a ≈1 eV higher BE than that of the P:LPSCl species, namely at 2148.1 eV, with a very broad FWHM of 2.9 eV, indicating undefined stoichiometry and/or the presence of more than one species (this will further on be referred to as “P:P_*x*_S_*y*_”). In studies showing P 2p spectra, the appearance/growth of higher these P:P_*x*_S_*y*_ feature upon LPSCl oxidation is accompanied by the simultaneous appearance growth of the S:P_*x*_S_*y*_ feature, indicating that they belong to the same species.^21,37,61^ The exact origin of the species assigned to the S:P_*x*_S_*y*_ feature is unknown, and it is surprising that they are not present in the pristine sheet-type separator (Figures S13b and S15b), which excludes the presence of impurities in the pristine LPSCl materials and any contamination/reaction that could have occurred during the solvent-based processing of the sheet-type materials, consistent with the report by Sedlmeier et al.^27^ In other studies, the presence of these species was explained by oxidation of LPSCl in the cell prior to electrochemical cycling.^12,61,62^ Thus, it cannot be excluded that the cell stack, experienced (chemically induced) degradation between cell assembly and the XPS measurements, leading to partially oxidized LPSCl. To aid further discussion, Table 1 summarizes the BE energy of the various discussed species as well as the FWHM values, and all S 1s *operando* spectra are displayed in Figure S12.

**Table 1:** BE and FWHM (in Parentheses) Values of the Different Species That have been Assigned to the Cl 1s, S 1s, and P 1s Spectra Shown in Figure 5; the Assigned Species Together with the Cl, S, and P Oxidation States (in Parentheses) are Given in the Second Column^a^

		BE (FWHM) [eV]			
core level	species (oxidation state)	BOT	*U*_CV_ = 4.10 V_Li_	*U*_CV_ = 2.50 V_Li_	*U*_CV_ = 5.50 V_Li_
Cl 1s	Cl:LPSCl (Cl −1)	2821.7 (1.9)	2822.1 (2.2)	2822.1 (2.2)	2822.1 (2.2)
	Cl:LiCl (Cl −1)	n/a	2820.0 (1.5)	2820.3 (1.5)	2820.3 (1.5)
S 1s	S:SO_4_ (S + 6)	2479.0 (1.8)	2478.1 (1.7)	n/a	n/a
	S:S_2_O_6_ (S + 5)	n/a	2477.3 (1.7)	2477.4 (2.0)	2477.0 (1.7)
	S:SO_3_ (S + 4)	2476.4 (1.5)	2476.2 (1.4)	n/a	n/a
	S:P_*x*_S_*y*_ (S 0/-1)	2471.6 (1.8)	2471.1 (1.9)	2471.0 (1.6)	2470.7 (2.0)
	S:LPSCl (S −2)	2470.0 (1.7)	2470.0 (1.7)	2470.2 (1.7)	2469.4 (1.7)
	S:Li_3_PS_4_ (S −2)	n/a	2468.3 (1.5)	2468.6 (1.3)	2468.0 (1.3)
P 1s	P:P_*x*_S_*y*_ (P + 5)	2148.1 (2.9)	2148.7 (2.6)	2148.8 (2.6)	2148.8 (2.6)
	P:LPSCl (P + 5)	2147.1 (1.9)	2147.2 (1.9)	2147.4 (1.9)	2147.4 (1.9)
	P:Li_3_PS_4_ (P + 5)	n/a	2145.6 (1.8)	2145.7 (1.8)	2145.7 (1.8)

a The spectra were collected *operando* either at BOT or during the OCV phase at the end of the first charge to *U*_CV_ = 4.10 V_Li_, at the end of the first discharge to *U*_CV_ = 2.50 V_Li_, or at the end of the second charge to *U*_CV_ = 5.50 V_Li_. The BEs are referenced to the BE of the MO_2_ peak that was set to 529.3 eV.

Figure 5b shows the XPS spectra at the end of the first charge to *U*_CV_ = 4.10 V_Li_, where new features appear in all three spectral regions, which clearly must derive from the oxidation of the LPSCl electrolyte. In the study by Tan et al.,^13^ the following oxidative decomposition reaction was proposed:

1 $${\mathrm{Li}}_{6}{\mathrm{PS}}_{5}\mathrm{Cl}\to \mathrm{LiCl}+\frac{1}{2}P_{2}S_{5}+\frac{5}{2}S+5{\mathrm{Li}}^{+}+5e^{-}$$

According to this mechanism, LiCl should be formed upon the oxidation of LPSCl. Thus, the new feature appearing in the Cl 1s region (light blue feature in Figure 5b) at a BE of 2820.0 eV, with a relatively narrow FWHM of 1.5 eV, can most likely be ascribed to LiCl (it will further on be referred to as “Cl:LiCl”), as observed in literature.^63^ Since the Cl 1s spectra were only acquired at BOT and at the end of the first charge to *U*_CV_ = 4.10 V_Li_, the onset potential for its formation cannot be determined.

In the high-BE S 1s region, the S:SO_3_ (dark pink) and S:SO_4_ (light pink) features have almost completely vanished at the end of the first charge (Figure 5b), and a new component with a BE in between those two components has appeared at 2477.3 eV (gray, FWHM = 1.7 eV). Figure S13a in the SI reveals that this happens gradually over the course of the first charge, initiating between ≈19–28% SOC (i.e., between *U*_CV_ ≈ 3.77–3.81 V_Li_). Based on the correlation by Yu et al.,^56^ the BE of this new feature would correspond to a sulfur oxidation state of S^5+^ and could thus indicate the presence of lithium dithionate (Li_2_S_2_O_6_; further on referred to as “S:S_2_O_6_”). A more quantitative analysis of the evolution of the relative amounts of the S:SO_3_, S:SO_4_, and S:S_2_O_6_ species (Figure S13a) reveals that the formation of the S:S_2_O_6_ species is accompanied by a roughly equimolar consumption of the S:SO_3_ and S:SO_4_ species, a reaction that apparently is driven by an increasing oxidative potential. A possible oxidation reaction that reflects the equimolar consumption of the sulfite and sulfate species (most likely sodium or lithium sulfite/sulfate) is given in the following, where M represents Na or Li:

$$M_{2}SO_{4}+M_{2}SO_{3}\to M_{2}S_{2}O_{6}+\frac{1}{2}O_{2}+2e^{-}+2M^{+}$$ 2

In aqueous media, dithionate forms at 0.6 V vs SHE (corresponding to ≈3.7 V_Li_) by the oxidation of H_2_SO_3_,^64^ which is surprisingly close to the onset of the formation of the S:S_2_O_6_ species observed in Figures S12 and S13a. The amount of the S:S_2_O_6_ species formed in the first charge remains constant until a potential of *U*_CV_ = 4.62 V_Li_ (95% SOC) is exceeded in the second charge (Figure S13c). As discussed above, both the sulfate and the sulfite species, and therefore also the dithionate species, seem to represent a small amount of contaminants in the sheet-type cathode and the NCM material (Figures S15 and S16), so that they are not considered in the following discussion of the oxidative decomposition of the LPSCl electrolyte.

The most evident change in the S 1s region near the S:LPSCl peak (beige) after the first charge to *U*_CV_ = 4.10 V_Li_ (Figure 5b) is the appearance of a new feature at 2468.3 eV (light green; FWHM = 1.5 eV), a BE that would typically be associated with Li_2_S.^26^ The fact that the appearance of the feature at 2468.3 eV is accompanied by the formation of a new feature in the P 1s region at 2145.6 eV (red colored peak; FWHM = 1.8 eV) provides some evidence that it may derive from an S and P-containing species instead, e.g., Li_3_PS_4_: the altered chemical environment of sulfur needs to be considered to rationalize the ≈1.5 eV lower BE of Li_3_PS_4_ compared to LPSCl. Generally, the negative charge around the sulfur is much better distributed among the lithium-rich crystal structure of LPSCl, and rather concentrated around the isolated Li_3_PS_4_ tetrahedra, whereby these slight changes in the polarization can already lead to a large shift in BE, as detailed in the publication by Dietrich et al.^65^ In principle, the formation of both Li_3_PS_4_ and Li_2_S could be justified by the oxidative LPSCl decomposition mechanism proposed by Morino et al.:^66^

3 $${\mathrm{Li}}_{6}{\mathrm{PS}}_{5}\mathrm{Cl}\to \mathrm{LiCl}+Li_{3}PS_{4}+Li_{2}S$$

However, the clearly potential-driven LPSCl decomposition cannot be explained by this formally potential-independent reaction. One possible explanation might be that the reaction is driven by the oxidative decomposition of one of the reaction products. Based on the lithium–sulfur battery literature, Li_2_S can indeed be readily oxidized in the commonly used aprotic electrolytes at reasonably low potentials (beyond ≈2.2–3.5 V_Li_);^38,67^ with its full oxidation to elemental sulfur given as

4 $${\mathrm{Li}}_{2}S\to 2{\mathrm{Li}}^{+}+2e^{-}+S$$

Hence due to the exceeded oxidative stability of Li_2_S at the given potentials, it can be excluded as a possible new species in the S 1s. Nonetheless, while eq 4 would be a plausible mechanism, the formation of elemental sulfur in the *operando* cell where the XPS-probed cathode surface is exposed to vacuum would result in a loss of sulfur,^68^ which is inconsistent with the observed constant S 1s signal in the BE region between 2466–2474 eV (open black symbols in Figure S13b). For the same reason, it would also be inconsistent with the reaction given by eq 1. Therefore, a more complex mechanism must be at play. Inspiration may again be drawn from the lithium–sulfur battery literature, which shows that the oxidation of Li_2_S proceeds via the intermediate formation of polysulfides, most commonly given as Li_2_S*_n_* (*n* = 1–8).^67,69^ With this in mind, eq 3 can be reformulated as

5 $$n\times {\mathrm{Li}}_{6}{\mathrm{PS}}_{5}\mathrm{Cl}\to n\times \mathrm{LiCl}+{n\times \mathrm{Li}}_{3}{\mathrm{PS}}_{4}+Li_{2}S_{n}+2\left(n-1\right)Li^{+}+2\left(n-1\right)e^{-}$$

The formal average oxidation state of the Li_2_S_*n*_ species then ranges from S^2–^ in Li_2_S (for *n* = 1 in eq 8), to an averaged S^–0.25^ in Li_2_S_8_ (for *n* = 8), which corresponds to two distinct sulfur oxidation states at the ends of the chain (S^1–^) vs in the middle (S^0^) once *n* ≥ 3.^67^ In the S 1s spectra, these Li_2_S_*n*_ chains are therefore likely not visible as an individual species, but are most probably included in the S:LPSCl and S:P_*x*_S_*y*_ features, and through the undefined stoichiometry and various chain lengths add to the high FWHM. Therefore, if the LPSCl oxidation reaction results in intermediate polysulfides (Li_2_S_*n*_) that have a higher oxidative stability than Li_2_S, no volatile sulfur component would be produced, explaining the constant S 1s signal throughout the second charge up to a potential of *U*_CV_ = 4.6 V_Li_ (Figure S13c). In summary, due to the oxidative instability in the probed potential window, as well as the fact that the reaction given by eq 5 would be consistent with the observed new Cl 1s, S 1s, and P 1s features at the end of the first charge to *U*_CV_ = 4.10 V_Li_ was well as with the constant S 1s intensity throughout the first charge, suggesting that the S 1s feature at 2468.3 eV can be ascribed exclusively to Li_3_PS_4_. For this reason, the S 1s feature at 2468.3 eV and the P 1s feature at 2145.6 eV will further on be referred to as “S:Li_3_PS_4_” and “P:Li_3_PS_4_”, respectively.

After the first discharge to 2.50 V_Li_, as shown in Figure 5c, no significant changes are observed in the XPS spectrum. However, after the second charge to 5.50 V_Li_ (Figure 5d), drastic changes can be noticed: (i) all features related to the LPSCl electrolyte in the S 1s, Cl 1s and P 1s spectra have decreased substantially (i.e., S:LPSCl, Cl:LPSCl, and P:LPSCl), whereby the S:LPSCl peak area decreased by ≈67% between 2.50 and 5.50 V_Li_ (Figure S13c); (ii) in the same potential window, the S:Li_3_PS_4_ peak area also decreased by ≈67% (Figure S13c) while the S:P_*x*_S_*y*_ increased ≈130% (Figure S13c); and, (iii) the overall area of the S:LPSCl, the S:Li_3_PS_4_, and the S:P_*x*_S_*y*_ peaks decreases significantly (Figure S13c), indicating a loss of sulfur from the sample. To rationalize these observations, the following mechanism is proposed, where in accordance with the literature Li_3_PS_4_, which is continuously being generated through the oxidation of LPSCl (eq 3), is further oxidized to elemental sulfur and P_*x*_S_*y*_ species.^13,66^ This is described by the following possible reaction pathways:

6 $$2Li_{3}PS_{4}\to {Li_{2}P}_{2}S_{6}+2S+4e^{-}+4Li^{+}$$

7 $$2Li_{3}PS_{4}\to P_{2}S_{5}+6{\mathrm{Li}}^{+}+{6e}^{-}+3S$$

which follows a complex oxidation pathway via Li_4_P_2_S_8_, Li_4_P_2_S_7_, and Li_4_P_2_S_6_ as intermediates.^21,70^ In addition, at high positive potentials, the proposed initially formed polysulfides (eq 5) will likely get oxidized:

8 $${\mathrm{Li}}_{2}S_{n}\to 2{\mathrm{Li}}^{+}+2e^{-}+n\times S$$

These three mechanisms lead to elemental sulfur, which under the vacuum conditions of the *operando* experiment would be expected to sublime.^68^ This is consistent with the fact that even though the area of the S:P_*x*_S_*y*_ feature increases toward the end of the second charge, the total area of all the S 1s peaks in the range of 2466–2474 eV decreases by ≈30% from BOT to 5.50 V_Li_ (Figure S13b and c), which we ascribe to the loss of the formed sulfur via sublimation. While the mechanism given by eqs 5–8 appears different from that proposed by Tan et al.^13^ (eq 1), it does follow from the here proposed sequence of reactions, i.e., the summing up eq 5, eq 7 (multiplied by *n*/2) and eq 8 yields eq 1.

At last, we would like to note that the BEs of all species in the Cl 1s and P 1s spectra stay constant, while those of the species in the S 1s spectrum slightly shift to lower BEs at high potentials (except for S:P_*x*_S_*y*_). We attribute this to the complex oxidation reactions and changing chemical environment, as further elaborated in Sections 3.1 and 3.3 in the SI.

### Operando S 1s Spectra

In this section, we want to analyze the exact onsets for the above-mentioned reactions and processes. For this, the S 1s *operando* spectra were plotted as a contour plot (Figure 6), where the spectra, after normalization to the area of the Ni 2p spectrum, were normalized to a global maximum value, i.e., the highest area-normalized intensity that was measured throughout the experiment, which enables a direct quantitative comparison between each scan. On the left side of the contour plot, the potential during the CV phase (*U*_CV_) and the SOC are given, starting with BOT from the top and going downward through the first charge, the first discharge, and the second charge. To facilitate the analysis, certain spectra were also selected and are shown alongside the contour plot on the right in panels a-h, where the different species are color-coded and marked as in Figure 5. First, we will first focus on the S:SO_4_ (2479.0 eV) and S:SO_3_ (2476.4 eV) species: when increasing the potential from BOT, the BE of both features remains constant without any shifts. At *U*_CV_ = 3.81 V_Li_ (28% SOC), the S:S_2_O_6_ species appear, accompanied by a decrease of the S:SO_4_ and S:SO_3_ signals. Upon the further charge to 4.10 V_Li_, the amount of the S:S_2_O_6_ species continues to grow, while S:SO_4_ and S:SO_3_ vanish, still retaining their BE from BOT.

**Figure 6:**
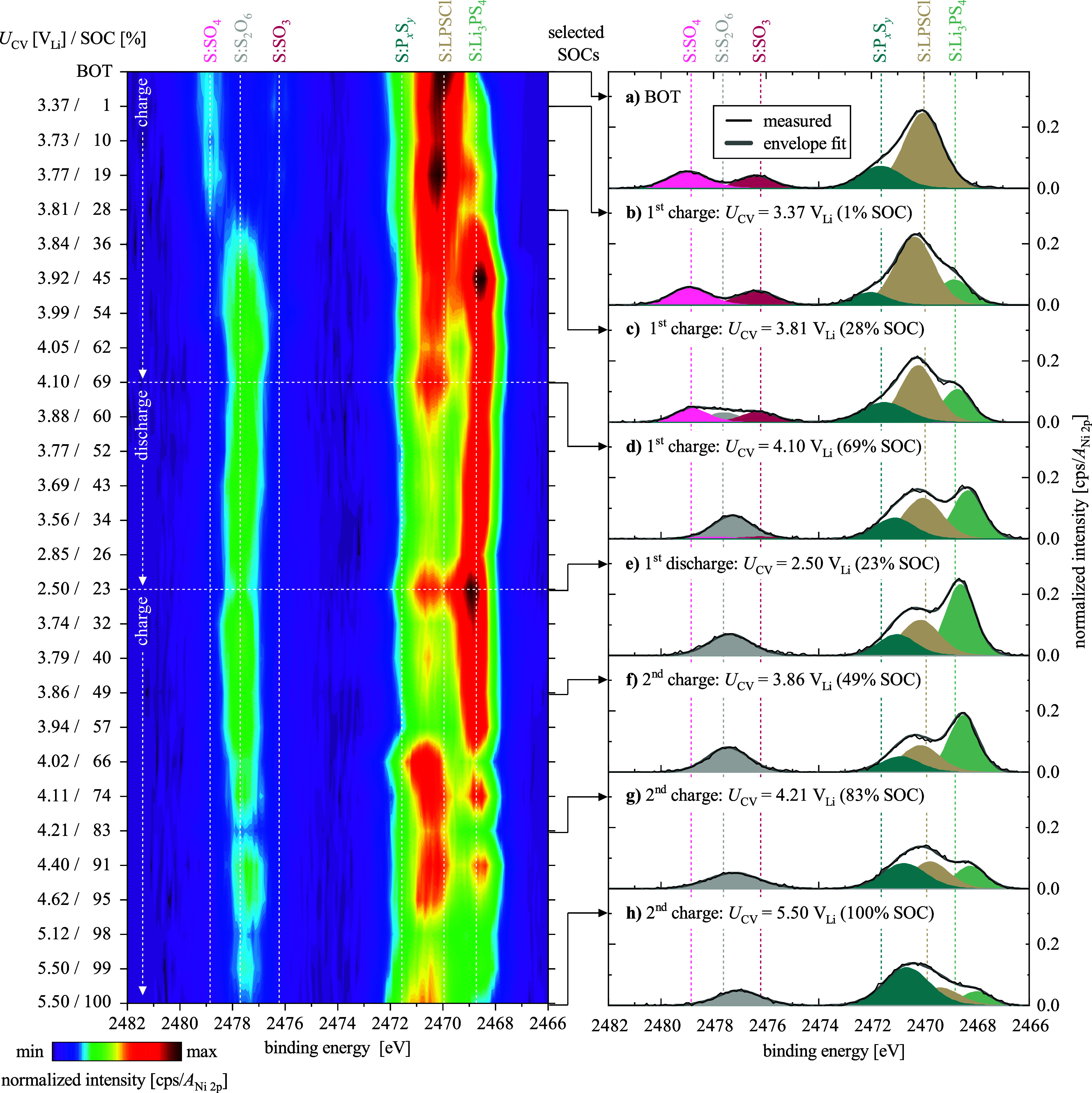
Left panel: Contour plot of the S 1s *operando* spectra over the course of the first charge to *U*_CV_ = 4.10 V_Li_, the first discharge to *U*_CV_ = 2.50 V_Li_, and the second charge to *U*_CV_ = 5.50 V_Li_ (from top to bottom), whereby both the *U*_CV_ and the respective SOC values are noted on the *y*-axis. The color scale at the bottom gives the Ni 2p-normalized intensity that was normalized also to a global maximum. Right panels (a–h): Detailed fits for the selected *U*_CV_/SOC values that are indicated on the right *y*-axis of the contour plot, namely (a) BOT, (b) 1%, (c) 28%, (d) 69%, (e) 23%, (f) 49%, (g) 83%, and (h) 100% SOC, with Ni 2p-normalized intensities. Shown are the as-measured signal (black lines), the envelope fit (gray lines), and the fits of the individual species (specified above the top panel) based on a Gaussian–Lorentzian blend of 30% on top of the Shirley background. All spectra are BE-corrected to the O 1s feature of the lattice oxygen in the layered oxide structure of the NCM (Figure S11).

When integrating the Ni 2p-normalized S 1s signals between 2474–2482 eV, the integral stays constant at 0.18 ± 0.02 (Figure S13a), and the formation of S:S_2_O_6_ signal seems directly linked to the decrease of the S:SO_4_ and S:SO_3_ signal, indicating the transformation of the two components into the dithionate. At the end of charge, and during the second charge, if *U*_CV_ ≥ 4.10 V_Li_, the intensity of S:S_2_O_6_ decreases slightly, which seems to mark the electrochemical stability limit for the S:S_2_O_6_ species. Upon comparison of these onset potentials with the reaction of observed BE shift and area of the RO_*x*_ species in the O 1s spectra (Figure 4), it seems likely that this was at least in parts, next to the decay of the carbonates in the C 1s, also due to the formation of the dithionate and the concomitant consumption of sulfates and sulfites (i.e., the earlier postulated species *S* could be sulfate/sulfite and S* would be dithionate).

The formation of the S:Li_3_PS_4_ interphase appears during the first polarization and can be seen from *U*_CV_ = 3.37 V_Li_ (1% SOC), as a broadening toward lower BEs in the contour plot, as well as in the normal plot in Figure 6b at 2468.9 eV. Although only a small step in SOC, the potential increases substantially, exceeding the electrochemical potential window of LPSCl (note the undefined potential of the In CE at BOT, as discussed before and along Figure S1).^12,39^ During charge, the area of S:Li_3_PS_4_ increases until *U*_CV_ ≤ 3.92 V_Li_ (45% SOC), and seems to form from S:LPSCl, which decreases simultaneously. Upon higher charge, the area of S:Li_3_PS_4_ starts to decrease, and that of S:P_*x*_S_*y*_ slightly increases. This is consistent with discharge, where the area of S:Li_3_PS_4_ increases once *U*_CV_ ≤ 3.9 V_Li_, as well as during the second charge, where the area of S:Li_3_PS_4_ remains constant (if one considers the integral at 2.50 V_Li_ as an outlier), yet again decreases if *U*_CV_ > 4.0 V_Li_. Hence, *U* ≈ 3.9 V_Li_ indicates the electrochemical stability limit for Li_3_PS_4_.

If *U*_CV_ ≥ 4.6 V_Li_, a decrease of −14% (EOT over area average during the second charge) of the overall sulfur intensity is observed until EOT. This is within the same potential range, where the applied potential during the CV-hold can no longer be kept in the OCV rest, and the potential decreases below the previous *U*_OCV_ (Figure 3). This seems to point in the direction of the reactions formulated by Koerver et al.,^21^ where Li_3_PS_4_ forms chains and then decomposes upon oxidation, which, for two Li_3_PS_4_ molecules, initially yields one S^0^ for the formation of Li_4_P_2_S_7_ (≡–12.5% sulfur) and two S^0^ for the continuous oxidation to Li_2_P_2_S_6_ (≡–25% sulfur, eq 6). While the observed detail reactions are in accordance with literature, the decrease of the total S 1s intensity appears slightly delayed. Likely, this can be explained by the rather complex mechanism for the formation of the Li_4_P_2_S_7_ and Li_2_P_2_S_6_ species, and/or simply because the sulfur is trapped and requires additional time to diffuse to the surface.

The findings of this study provide crucial insight into the stability limits of the SE components and decomposition products. Hence, the detected stability limits for the components are summarized in Table 2, and the impact of the adapted stability limits will be the focus of a follow-up study.

**Table 2:** Electrochemical Stability Limit of Different S 1s Species Determined from the Data in Figure 6 ^a^

species	formation *U*_CV_	decomposition *U*_CV_
S:SO_4_	CAM	3.7 V_Li_
S:S_2_O_6_	3.7 V_Li_	4.0 V_Li_
S:SO_3_	LPSCl	3.7 V_Li_
S:P_*x*_S_*y*_	LPSCl	4.6 V_Li_
S:LPSCl	LPSCl	3.4 V_Li_
S:Li_3_PS_4_	3.1 V_Li_	3.9 V_Li_

a The potential refers to the CV phase, and the CV vs. OCV potential comparison is detailed in Figure 3.

To summarize, after the initial polarization, Li_3_PS_4_ is formed (eq 5), which, together with the polysulfides, could be considered a stable interphase and serve as the CEI. This interphase is stable until *U*_CV_ = 3.9 V_Li_, where Li_3_PS_4_ degrades, forming further polysulfides (eqs 6+[Disp-formula eq7]). If the potential is then increased beyond *U*_CV_ = 4.6 V_Li_, all S-based components, as well as the CAM, degrade (eq 8). Hence, if a cell with LPSCl is operated to an upper potential cutoff of *U*_CV_ < 3.9 V_Li_, the cycling stability and capacity retention might be better than if the cutoff is increased to/above 3.9 V_Li_. Thus, the results of this study actively contribute to a longer lifetime of battery cells, and thus aim toward a more sustainable operation of battery cells.

The observations are graphically summarized in Scheme 1, where the electrochemistry is correlated to the XPS observations at OCV and 5.50 V_Li_. The composite cathode is sketched using an NCM active material particle (dark gray), C65 (black) and SE (yellow). The electrochemistry is plotted on the left and shows the cycled potential as a function of the normalized capacity. This further illustrates how the XPS spectra for O 1s and S 1s change drastically from BOT at OCV to the end at 5.50 V_Li_.

**Scheme 1:**
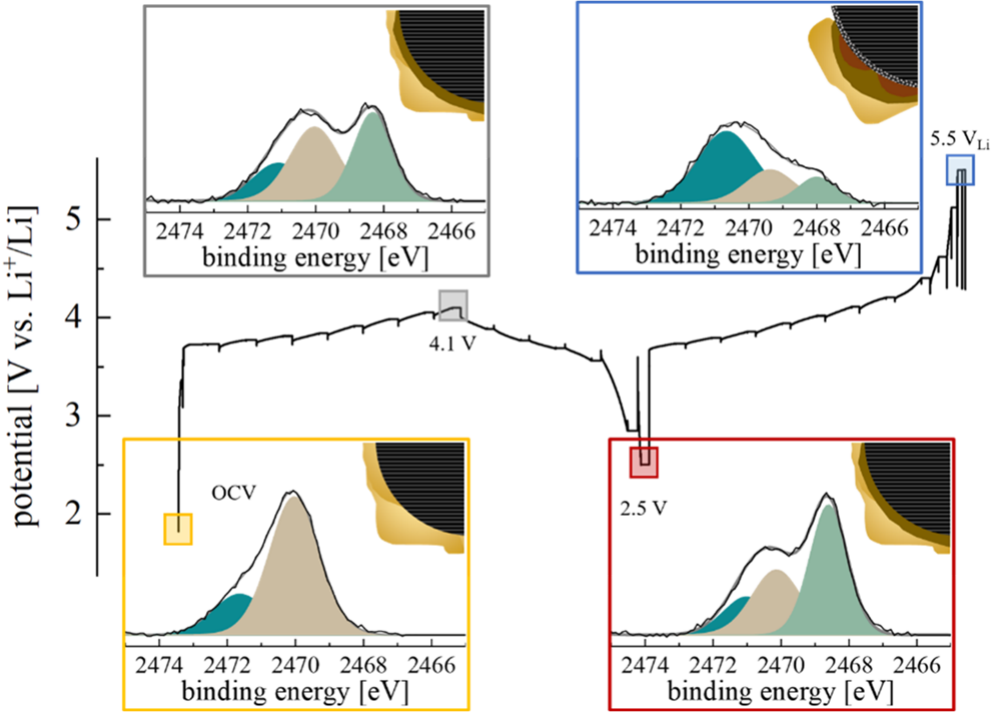
Phenomenological Graphical Summary of the Observed Key Findings Regarding the SE Redox and the Stability of the Active Material in an ASSB-Type *Operando* XPS Cell Configuration

## Conclusions

The stability limit of an LPSCl-based composite cathode with a Ni-rich NCM was studied using a novel *operando* XPS setup, and the formation of a CEI, as well as the oxidative behavior of the argyrodite was elaborated, as a function of the applied potential. For this, an ASSB setup, inspired by a state-of-the-art pouch cell design, was developed, and could be unambiguously correlated to the former, with an average overpotential of only ≈40 mV between the *C*/10 constant current and OCV.

Spectroscopically, the setup was validated by analyzing the O 1s spectra of the Ni-rich cathode, where the onset of oxygen release was detected at 74% SOC after the CV phase at 4.10 V_Li_, which was in accordance with literature results. Here, signs indicating the presence of complex reactions with miscellaneous oxygen-containing species highlighted the role of the SE, where contaminants and side products were observed to degrade at the same potential. Additionally, upon initial polarization of the cell, the SE disintegrates, and LiCl and Li_3_PS_4_ are found, forming a CEI. Li_3_PS_4_ is stable until reaching ≈3.9 V_Li_, where it decomposes to complex polysulfides that form elemental sulfur if *U*_CV_ > 4.6 V_Li_. While the found mechanism is overall in accordance with literature, it adds new levels of depth and elucidates the detailed reactions based on potential and SOC. In conclusion, this study provides insights into the mechanistic behavior of LPSCl. With the determined stability limits of the individual components, the longevity of ASSB cells can be improved, and the degradation of the LPSCl can be minimized by optimizing the potential window during future studies.

## Supplementary Material



## Data Availability

The data that support the findings of this study are available from the authors upon reasonable request.

## Appendix

The used abbreviations throughout this study are summarized in Table 3.

**Table 3:** Summary of Abbreviations Used throughout This Publication

abbreviation	meaning
ASSB	all-solid-state battery
BE	binding energy
CAM	cathode active material
CC	constant current
CE	counter electrode
CEI	cathode/electrolyte interphase
CV	constant voltage
EDX	energy dispersive X-ray spectroscopy
FWHM	full width at half maximum
LPSCl	Li_6_PS_5_Cl
SOC	state of charge
OCV	open circuit voltage
SE	solid electrolyte
SEM	scanning electron microscopy
WE	working electrode
XPS	X-ray photoelectron spectroscopy

## Notes

Discussion of the counter electrode potential determination (Figure S1); state of charge dependent potential relaxation during data collection (Figure S2); potential relaxation between constant voltage and open circuit voltage phase (Table S1); cyclic voltammetry measurement of a solid electrolyte/carbon model electrode (Figure S3); comparison of the electrochemical performance to different benchmark cells (Figure S4); analysis of possible beam damage effect (Figure S5); O 1s on surface vs under current collector (Figure S6); nickel Ni 2p spectra in *operando* (Figure S7); background-corrected, normalized Ni 2p at selected SOCs (Figure S8); integrated areas of the Ni 2p core level (Table S2); binding energy of Ni 2p_3/2_ (Figure S9); carbon C 1s spectra in *operando* (Figure S10); binding energy of surface-oxygen species and layered oxide in O 1s before and after correction (Figure S11); discussion of the RO_*x*_ area (Table S3); sulfur S 1s spectra in *operando* (Figure S12); extracted areas of S-containing species (Figure S13); binding energy of S-containing species (Figure S14); XPS reference sample measurements at ALS (Figure S15); XPS reference sample measurements using Al K_a_ (Figure S16); comparison to S 1s BE to literature (Table S4) (PDF)
