# Supplementary material for: Exploring the Electrochemical Stability Window of an All-Solid-State Composite Cathode via a Novel Operando Tender XPS Setup
Source: ACS Appl Mater Interfaces. 2025 Jun 18;17(26):38571–86. doi: 10.1021/acsami.5c01672 (PMC12232285; doi:10.1021/acsami.5c01672)
Supplement: Supplementary file 1 [file am5c01672_si_001.pdf]

Supporting Information

for

Exploring the electrochemical stability window of an all-solid-state composite cathode via a novel *operando* tender XPS setup

Rebecca Wilhelm[1,*,=], Robin Schuster[1,2,=], Tobias Kutsch[1,2], Simon Qian[1], Johannes Mahl[3], Tim Kratky[4], Johannes Wandt[5,6], Ethan J. Crumlin[3,*] and Hubert A. Gasteiger[1]

[1]Technical University of Munich, TUM School of Natural Sciences, Department of Chemistry and Catalysis Research Center, Chair of Technical Electrochemistry, Lichtenbergstraße 4, 85748 Garching, Germany

[2]TUMint·Energy Research GmbH, Lichtenbergstraße 4, 85748 Garching, Germany

[3]Advanced Light Source, Lawrence Berkeley National Laboratory, Berkeley, California 94720, United States; Chemical Sciences Division, Lawrence Berkeley National Laboratory, Berkeley, California 94720, United States

[4]Technical University of Munich, TUM School of Natural Sciences, Department of Chemistry and Catalysis Research Center, Physical Chemistry with Focus on Catalysis, Lichtenbergstraße 4, 85748 Garching, Germany

[5]BMW AG, 80788 Munich, Germany

[6]University of Agder, 4879 Grimstad, Norway

[*]Co-corresponding authors: Rebecca.Wilhelm@tum.de and ejcrumlin@lbl.gov

[=]These authors contributed equally.

Supporting Information

Table of Contents

1. Supplementary information for the electrochemical measurements S02
2. Analysis of probing depth and evaluation of beam damage effects S09
3. Operando reference spectra for Ni 2p, C 1s, O 1s, and S 1s S12
4. XPS measurements of reference samples S22

References S24

1. Supplementary information for the electrochemical measurements

1.1. Determining the potential of the indium CE

For practical reasons, a non lithiated indium foil was chosen as the CE for the *operando* cell setup. The consequence is, that the CE potential is undefined before the indium is lithiated to a certain degree, namely until an In_1Li_1 alloy phase is formed at the indium surface region adjacent to the SE, which then provides a defined and stable potential of +0.62 V vs. Li^+/Li (V_{Li}).[1] To determine to which degree the indium metal CE in the *operando* cell must be lithiated to reach +0.62 V_{Li}, a separate experiment was conducted as depicted in Fig. S1a: A prelithiated indium foil ("InLi", with 14 mm diameter, prepared as described previously),[2]an LPSCl sheet-type separator, and the same type of indium foil that was used as CE for the *operando* cell ("In", with 13 mm diameter, ≈ 250 µm thick, 260 mg indium) were assembled in a pouch cell setup and compressed at 30 MPa (compare to Sedlmeier et al.).[3] The indium foil was then lithiated stepwise, applying segments of 0.77 µA cm^{-2} for 1 h followed by a 1 h OCV period, until the OCV of the cell would reach 0 V. As the InLi electrode has a known potential of +0.62 V_{Li}, a cell voltage of 0 V implies that the In electrode has also reached a potential of +0.62 V_{Li}. As shown in Fig. S1 (black line), the OCV of the cell reaches ≈ 0 V after the In electrode was lithiated with 5.4 µAh cm^{-2}, corresponding to a 0.01 at% lithiation of the indium foil. This suggests that after a lithiation charge corresponding to a lithium content of 0.01 at% in the indium foil, a stable potential of +0.62 V_{Li} can be achieved.

For the *operando* XPS cell shown in the main text (Fig. 3), where the above discussed indium foil was also used as a non-lithiated CE, a lithium content of 0.01 at% would be achieved upon a lithiation charge of 5.4 µAh cm^{-2}. An inspection of the voltage versus time profile of the *operando* cell (black line in Fig. S1b) during the initial steps of its first charge indicates a very steep increase of the cell voltage in the first charge step (CC charge at *C*/10 for 5 min) during which the lithiation charge of the indium CE was

≈ 2.3 µAh cm^{-2} (green line and right y-axis in Fig. S1b). A large part of this initial cell voltage increase must be caused by the rapid decrease of the indium CE potential as the indium is being lithiated; for example, upon a lithiation of the indium electrode in Fig. S1a by 2.3 µAh cm^{-2}, its potential decreases by almost 1 V (i.e., from ≈ 1.8 V_{Li} to ≈ 0.8 V_{Li}), from BOT until $t \approx 8$ h. Therefore, in this first charge segment, charging the composite cathode of the *operando* cell to ≈ 1 % SOC, the cathode potential cannot be determined from the cell voltage (indicated by the gray shaded area). However, in the second charge segment of the *operando* cell, the above estimated lithiation charge of the indium CE of 5.4 µAh cm^{-2} is reached within a short time (marked by the vertical red dotted line), namely after a cathode SOC of ≈ 2 %. From this point on, one would expect that the indium CE has assumed a defined potential of +0.62 V_{Li}, so that any cell voltage changes are now solely due to changes in the cathode potential. This is consistent with the nearly constant cell voltage upon a charge of > 2 % SOC, as for the cathode potential versus SOC profile of the here used Ni-rich CAM has a very small slope below ≈ 20 % SOC. In summary, the conversion of the *operando* cell voltage to V_{Li} is correct once the SOC of the cathode exceeds ≈ 2 % SOC in the first charge. This therefore allows a rigorous correlation between the acquired XPS spectra of the composite cathode with the cathode potential.

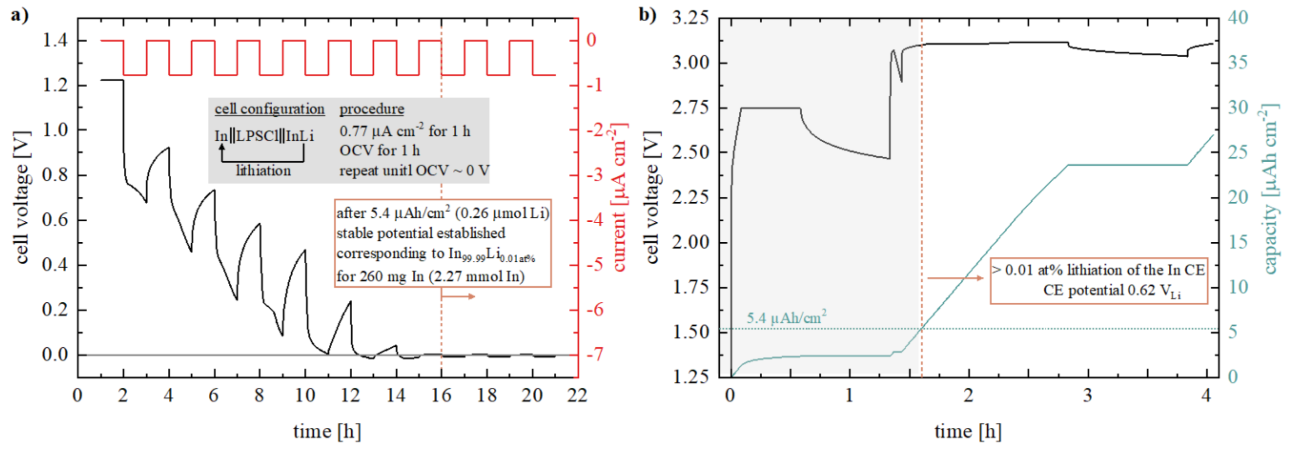

Figure S1: a) Cell voltage of an In/LPSCl/InLi pouch cell during sequential lithiation of the indium electrode (steps of 0.77 µA cm^{-2} lithiation for 1 h, followed by 1 h OCV) in order to determine the required amount of charge to obtain a stable potential of the indium electrode of +0.62 V_{Li}. **b)** Voltage versus time profile of the *operando* cell for the first few charging step sequences during the first charge of the cell (black line, taken from Fig. 3 in the main text). The green line corresponds to the accumulated lithiation charge applied to the indium CE (plotted versus the right *y*-axis), with the red dotted line marking the charge beyond of which a stable indium CE potential of +0.62 V_{Li} is expected.

1.2. Potential relaxation during the OCV phases during charge/discharge of the operando cell

After the CV hold at a given potential, the cell was put in OCV for ≈ 5 min prior to the start XPS data collection, where the majority of the potential relaxation occurs. This is shown in Fig. S2, which delineates the total potential drop during the entire OCV phase (sum of beige and green bars) from that that occurred prior (green bars) and during the XPS data acquisition (beige bars). For most measurement points, the OCV relaxation during XPS data acquisition is below ≈ 50 mV. Consequently, the SOC during XPS data acquisition can be considered to be relatively well-defined and the chemical state is equilibrated. However, at the SOC of ≈ 1 % during the first charge (left-most set of bars) a relatively large potential drop during the measurement phase was observed (≈ 160 mV), which could be related to: (i) a large charge transfer resistance (R_{CT}) of the NCM at very low SOCs, (ii) an ongoing formation of a CEI upon reaching anodic the stability limit of the SE, and/or (iii) a variation of the CE potential that is poorly defined in this first charge sequence (Fig. S1). Additionally, at high cathode potentials of $U_{CV} > 4.60$ V_{Li}, the OCV relaxation during XPS data acquisition is on the order of ≈ 75 - 175 mV, which is likely due to the slow charge transfer kinetics of the CAM at high SOC and further influenced by significant electrolyte decomposition reactions at high potential ($U_{CV} > 4.6$ V_{Li}).

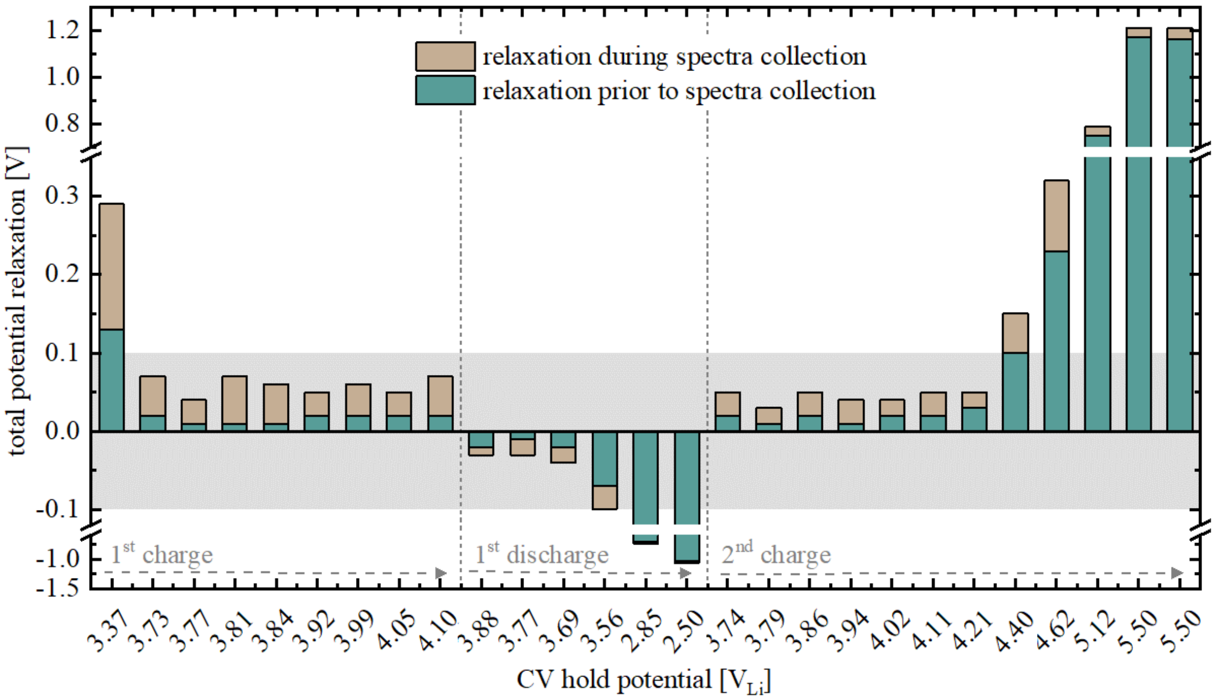

Figure S2: Potential relaxation between a prior CV hold step (x-axis) and the subsequent OCV phase, delineated into the OCV relaxation during the ≈ 5 min time prior to spectra collection (green bars) and during the XPS data acquisition (beige bars).

Table S1*:* Supplementary data for the potential relaxation during the OCV phases. The table lists the SOC of the *operando* cell that was reached after a given CV hold potential (U_{CV}), the OCV at the begin of the XPS data acquisition ($OCV_{XPS\text{-}start}$), the change in OCV during the XPS data acquisition (ΔOCV_{XPS}), and the OCV at the end of the XPS data acquisition and thus the end of the OCV period, which is referred to as U_{OCV} both here and in the main text. All potentials refer to the cathode potential vs. Li^+/Li [V_{Li}], except for the potentials at BOT and 1 % SOC (section 1.1 of the SI), marked by quotation marks.

SOC %	U_{CV} [V_{Li}]	$OCV_{XPS\text{-}start}$ [V_{Li}]	ΔOCV_{XPS} [V]	U_{OCV} [V_{Li}]
0% (BOT)	-	-	-	"1.22"
1st charge to 4.10 V_{Li}				
1	"3.37"	"3.24"	-0.15	"3.08"
10	3.73	3.71	-0.05	3.66
19	3.77	3.76	-0.03	3.73
28	3.81	3.80	-0.06	3.74
36	3.84	3.83	-0.05	3.78
45	3.92	3.90	-0.03	3.87
54	3.99	3.97	-0.04	3.93
62	4.05	4.03	-0.03	4.00
69	4.10	4.08	-0.05	4.03
1st discharge to 2.50 V_{Li}				
60	3.88	3.90	0.01	3.91
52	3.77	3.78	0.02	3.80
43	3.69	3.71	0.02	3.73
34	3.56	3.63	0.03	3.66
26	2.85	3.56	0.03	3.59
23	2.50	3.53	0.04	3.57
2nd charge to 5.50 V_{Li}				
32	3.74	3.72	-0.03	3.69
40	3.79	3.78	-0.02	3.76
49	3.86	3.84	-0.03	3.81
57	3.94	3.93	-0.03	3.90
66	4.02	4.00	-0.02	3.98
74	4.11	4.09	-0.03	4.06
83	4.21	4.18	-0.02	4.16
91	4.40	4.30	-0.06	4.25
95	4.62	4.39	-0.09	4.30
98	5.12	4.37	-0.04	4.33
99	5.50	4.33	-0.04	4.29
100	5.50	4.34	-0.06	4.29

1.3. LPSCl oxidation in a SE/C model electrode

To confirm the observed oxidation behavior of the LPSCl SE, it was investigated using a SE/C65 model electrode in order to exclude possible contributions from the NCM material. The model electrode consists of 79 wt% LPSCl, 19 wt% C65, and 2 wt% PIB and was assembled analogously to the *operando* cell stack (see experimental section in the main text), except that a prelithiated InLi CE was used. The potential of the as-assembled SE/C electrode (referenced to Li^+/Li) was scanned at 0.05 mV s^{-1} from its initial OCV to 4.10 V_{Li}, then down to 2.50 V_{Li}, and finally up to 5.50 V_{Li}, using the same potential limits as those used in the testing procedure of the *operando* cell.

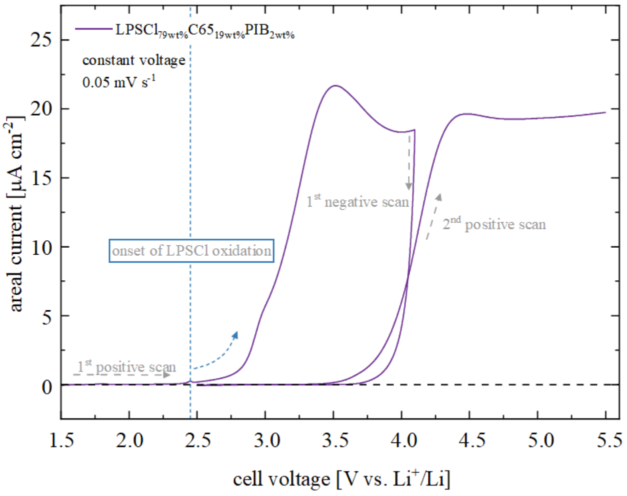

Figure S3: Current versus potential response of an SE/C65 model electrode subjected to constant voltage sweeps (0.05 mV s^{-1}), first from its initial OCV to 4.10 V_{Li}, then back to 2.50 V_{Li}, and finally back up to 5.50 V_{Li}. The cell consists of an SE/C65 working electrode and an InLi CE, separated by an LPSCl SE.

The resulting current versus potential profile is shown in Fig. S3. During the first positive-going scan, the current starts to increase significantly at a potential above ≈ 2.45 V_{Li}, indicating the onset of LPSCl oxidation. As observed during the measurement of the *operando* cell, very little current is obtained during the first negative-going scan (corresponding to the first discharge of the *operando* cell), indicating a quasi-stable state. Only upon further increasing the potential of the SE/C electrode in the second positive-going scan to 5.50 V_{Li}, the LPSCl decomposition commences again at potentials exceeding ≈ 3.5 V_{Li}. Over the course of these three potential scans, 0.32 µAh of oxidative charge was obtained, which corresponds to a ≈ 3.2 nm thick decomposed SE layer when assuming a specific oxidation capacity of 499 mAh g_{LPSCl}^{-1} (acc. to Eq. S1, proposed by Tan et al.).[4] and an average density of the solid components corresponding to that of the LPSCl (1.85 g cm^{-3}).

$$Li_6PS_5Cl \rightarrow LiCl + \frac{1}{2}P_2S_5 + \frac{5}{2}S + 5Li^+ + 5\,e^- \qquad (S1)$$

We anticipate the decomposed layer in the SE/C electrode to be slightly thicker because the calculation assumes complete contact between the entire C65 surface and the SE. However, this assumption may not entirely accurate due to the high number of micro pores in the C65, which may prevent a complete coating of the C65 surface with the SE, in which case the here assumed C65 surface area (based on the C65 BET area) would be larger than the actual contact area between the C65 and the SE, so that the actual thickness of the decomposed SE layer would be correspondingly larger.

1.4. Validation of the capacity normalization

The theoretical, i.e., full delithiation capacity of the $LiNi_{0.85}Co_{0.10}Mn_{0.05}O_2$ (NCM) is 275 mAh g_{NCM}^{-1}, which is very closely reached for an NCM with the same composition for a slow CC charge up to 5 V_{Li} in a liquid-electrolyte cell (namely ≈ 267 mAh g_{NCM}^{-1}).[5] In order to compare this with an LPSCl SE-based cell with the same NCM, a cell with a sheet-type cathode was prepared in the same manner as for the *operando* cells (see experimental section in the main text), except that i) a ≈ 10-fold higher cathode loading (≈ 18.9 mg_{NCM} cm^{-2}, corresponding to ≈ 5.2 mAh cm^{-2}) was used in order to minimize weighing errors and that ii) the cell stack was assembled in a pouch cell to guarantee a perfectly homogeneous compression of 30 MPa.

The first CC charge of this pouch cell ("PC-C (5.2 mAh cm^{-2})") at a rate of *C*/100 to 5.50 V_{Li} is depicted by the green line in Fig. S4a, yielding a specific charge capacity of ≈ 262 mAh g_{NCM}^{-1}, which is very close to the value obtained for a liquid-electrolyte cell and within 5 % of the theoretical first-charge capacity of 275 mAh g_{NCM}^{-1}. For the very thin, low-loaded cathode used in the *operando* experiments (≈ 1.1 - 2.0 mg_{NCM} cm^{-2}, corresponding to ≈ 0.3 - 0.5 mAh cm^{-2}), however, the quantification of the cathode loading has an estimated error of ≈ 10 %, and a pouch cell assembled with a cathode with a nominal loading of 1.45 mg_{NCM} cm^{-2} ("PC-A (0.4 mAh cm^{-2})") reaches a first-charge capacity of only ≈ 245 mAh g_{NCM}^{-1} at *C*/100 to 5.50 V_{Li} (blue line in Fig. S4), a difference which we ascribe to an error in the NCM loading quantification. The loading error can be exacerbated when the aluminum current collector (Al-CC) is removed, as is required for the *operando* cell measurements (Fig. 1, main text), since a minor fraction of the cathode material sticks to the aluminum foil; while this could in principle be accounted for by subtracting the mass of the removed aluminum foil, one must consider that for such thin electrodes also the ratio of SE and NCM could be changed slightly, introducing an additional error when trying to quantify the NCM content of the composite cathode. The first-charge capacity of a thin, low-loaded cathode after removal of the aluminum foil when measured in the pouch cell configuration is shown for a such prepared cathode with a nominal loading of 1.82 mg_{NCM} cm^{-2} ("PC-B

(0.5 mAh cm^{-2}), Al-CC removed”), reaching a first-charge capacity of only ≈ 233 mAh g_{NCM}^{-1} at *C*/100 to 5.50 V_{Li} (orange line in Fig. S4).

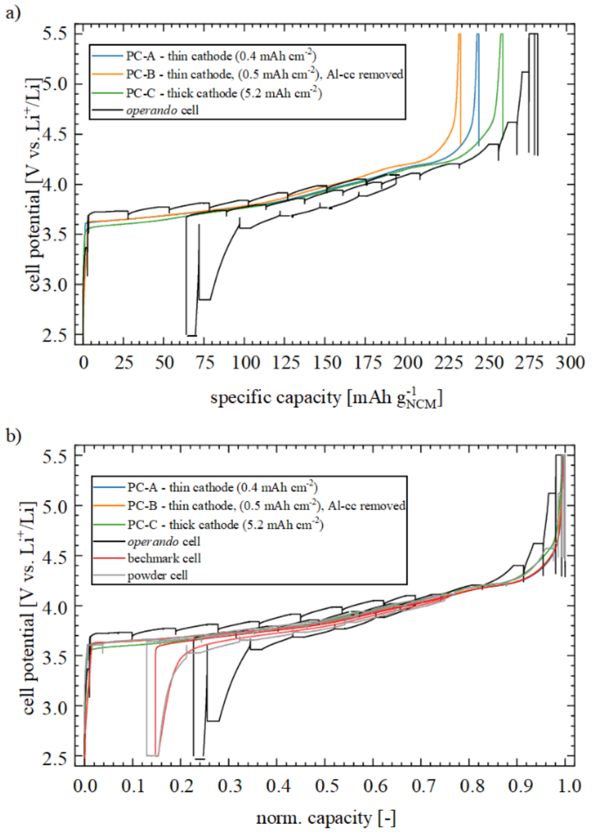

Figure S4: a) Voltage profiles versus nominal NCM specific capacity (based on cathode weight measurements) of the *operando* cell during two charge/discharge cycles with intermittent CV-hold and OCV periods (black line; same data as shown in Fig. 3 of the main text) compared to those of pouch cells (PC) based on identically prepared sheet-type cathodes that were charged to 5.50 V_{Li} at a rate of *C*/100 under a compression of 30 MPa. Cell “PC-C (5.2 mAh cm^{-2})” (green line) had a high-loaded, thick composite cathode for which the NCM loading can be determined rather precisely. Cell “PC-A (0.4 mAh cm^{-2})” (blue line) had a low-loaded, thin composite cathode, for which the NCM loading error is estimated to be roughly ±10 %. Cell “PC-B (0.5 mAh cm^{-2})” (orange line) had a low-loaded, thin composite cathode from which the Al foil current collector had been removed (as was the case for the *operando* cell), which introduces larger uncertainties in the NCM loading. **b)** Voltage profiles normalized to the maximum nominal NCM specific capacity values in panel a, together with the benchmark cell (Figure 3, red) and a powder-based cell (gray).

In Fig. S4b, the potential profiles of the three pouch cells are normalized to the respective maximum achieved capacities: the fact that in this case the voltage profiles of all three cells superimpose rather perfectly (green, orange, and blue lines) indicates that the differences between in the voltage profiles versus the nominal NCM specific capacity in Fig. S4a are due to errors in the quantification of the exact NCM mass for the low-loaded electrodes. Furthermore, the good correlation between the powder cell and the benchmark cell, shows the good comparability to more widespread powder-based setups and validated the approach for the SOC determination.

The charge/discharge profiles of the *operando* cell that were investigated by *operando* XPS at the ALS are shown by the black lines in Fig. S4, whereby in this case intermittent CV-hold and OCV periods were applied (same data as shown in Fig. 3, main text). Here, the specific charge capacity up to 5.5 V_{Li} in the second cycle of ≈ 283 mAh g_{NCM}^{-1} slightly exceeds the theoretical capacity of 275 mAh g_{NCM}^{-1} (Fig. S4a). However, when plotted capacity-normalized in Fig. S4b, the OCV points of the charge/discharge curves very closely agree with the *C*/100 voltage profiles obtained in the pouch cells, except for normalized capacities of ≥ 95 %. Owing to this reasonably good agreement, the SOC values for the *operando* cell measurement were based on the SOC values that are indicated by the OCV points in Fig. S4b.

2. Analysis of probing depth and evaluation of beam damage effects

To select an optimized incident beam energy at the ALS, two main considerations were considered, namely that the chosen beam energy was (i) high enough to probe the phase changes in NCM and LPSCl, and (ii) as low as possible to minimize the risk of beam-induced spectral changes as well as reducing the attenuation of the core level cross-sections which otherwise would lead to longer collection times to obtain the same signal to noise ratio. The inelastic mean free path for the NCM (λ_{NCM}) can be calculated using the TPP2m equation,[6,7] and is estimated to be $\lambda_{NCM} \approx 6$ nm. Since the escape depth probability w scales as a function of $w \sim \exp(-z/\lambda)$, where z denotes the depth through which the electrons travel, using an incident beam energy of 4 keV is sufficient to probe the LPSCl, as well as the layered oxide below the rocksalt phase after cycling to high potentials.

To evaluate the stability of the working electrode under the X-ray beam at the chosen conditions, preliminary tests at the ALS were performed, where an as-coated, uncompressed sheet-type cathode was glued with the aluminum current collector foil side onto a double-sided carbon tape that was attached onto the cell's front plate (see Fig. 1), so that the composite cathode coating would be exposed to the X-ray beam. The cell was then transferred into the measurement chamber and the XPS data acquisition procedures were first optimized to ensure a good signal-to-noise ratio for the *operando* data collection, moving to a new spot on the sample for each spectral region. Subsequently, the beam was moved to a new spot, and 10 consecutive scans were recorded for the O 1s region, each with an acquisition time of ≈ 1 min (upper panel of Fig. S5, scans #1 - #10). Upon moving to a new spot, a total of 4 spectra each were acquired alternatingly in the S 1s and C 1s regions (spectra #1 - #4 in the middle and lower panel of Fig. S5), each with an acquisition time of ≈ 1 min. The essentially perfect overlap of the consecutively acquired spectra in Fig. S5 indicates the absence of any beam damage. The Ni 2p spectra were omitted, because a previous *operando* XPS study had already shown the stability of NCM under the X-ray beam

for an even higher beam energy,[7] so that we considered the NCM material to be stable under our measurement conditions and focused on the LPSCl.

After probing the composite cathode for beam-induced changes on the minute-scale, the now fully-optimized procedure was applied, and the sample was irradiated for another $t \approx 50$ min. As the pass energy was changed for the optimized procedure, the features are somewhat more narrow, but the shape of the cps in the core level remain unchanged (data not shown). Hence also here, beam damage on a larger time scale can be excluded.

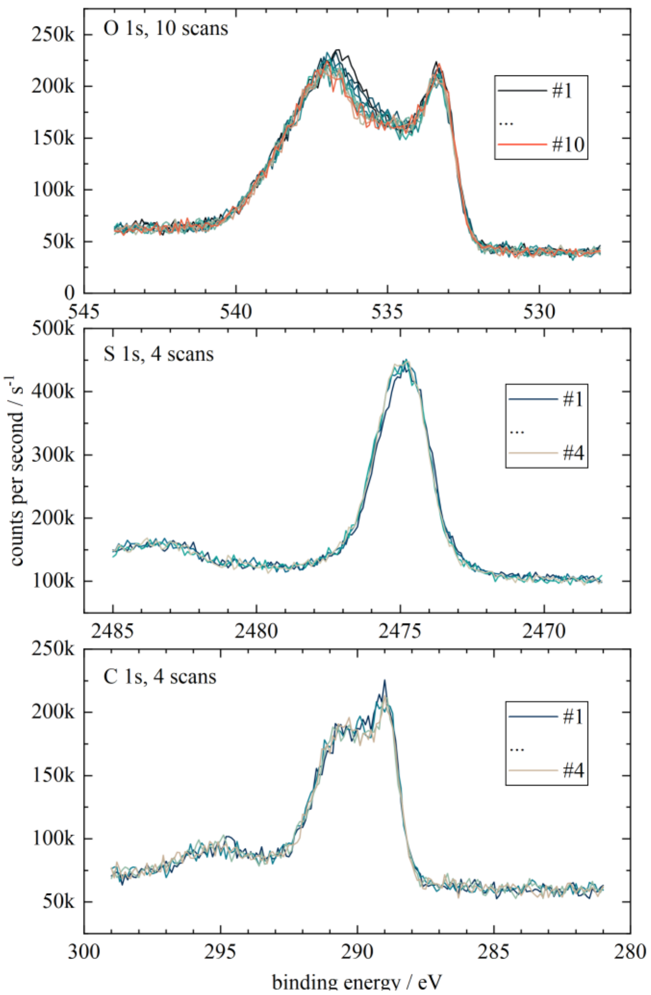

Figure S5: O 1s, S 1s, and C 1s spectra, recorded on a pristine cathode using a 4 keV X-ray beam. The upper panel shows 10 consecutive O 1s spectra recorded on a new sample spot (≈ 1 min/spectrum). The middle and lower panel show 4 sets of alternately acquired S 1s and C 1s spectra on a new sample spot (≈ 1 min/spectrum). Note that no BE corrections have been applied for these spectra, i.e., the *x*-axis energy scale is as-measured.

It should be mentioned that the general shape of the O 1s spectra here differs from those shown in Fig. 4 of the main text. This follows an observation we also made for O 1s spectra acquired with our in-house XPS, where small differences in the O 1s spectra were observed depending on the side of the composite cathode on which the spectra were acquired: i) For the measurements shown in Fig. S5, the surface of the

as-coated composite cathode was analyzed by XPS, i.e., the surface of the composite cathode that is exposed after its coating onto the aluminum current collector (Al-CC), illustrated by the upper sketch in Fig. S6 and labeled a “as-coated surface”. ii) On the other hand, for the *operando* XPS measurements, the cathode surface from which the XPS spectra were acquired is the surface that is generated upon the removal of the Al-CC foil (Fig. 1, main text), shown by the lower sketch in Fig. S6 and labeled as “surface under the Al-CC”. As elaborated in the main text, during the removal of the Al-CC foil, also minor parts of the composite cathode itself remained on the removed Al-CC foil, leading to the exposure of a fresh cathode surface. As can be seen in Fig. S6, compared to the O 1s spectrum acquired from the “as-coated surface” (green line), the O 1s spectrum acquired from the “surface under the Al-CC” (blue line) has a slightly more resolved separation between the high-BE peak ascribed to CAM surface contaminants and the low-BE peak from the oxygen in the layered oxide phase of the NCM (see main text for details). This is likely due to a lower extent of surface contaminants on the “surface under the Al-CC”, which was exposed to the glovebox atmosphere for only very short time during cell assembly (minutes), in contrast to the long-time exposure experienced by the “as-coated surface” during the electrode coating and drying process (hours). Hence, also *operando* spectra acquired at the ALS yielded a better separation between the two O 1s peaks (Fig. 4, main text) compared to the O 1s spectra recorded from the “as-coated surface” in Fig. S5.

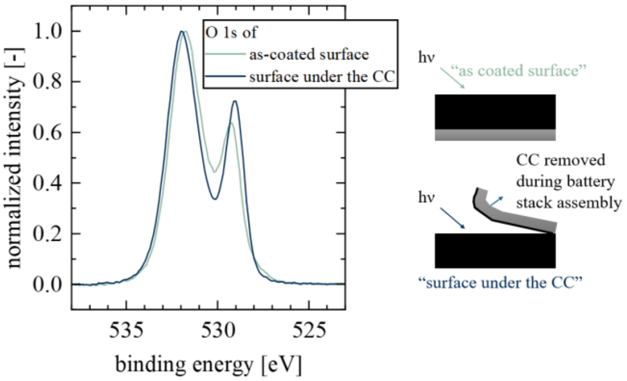

Figure S6: O 1s spectra, recorded on pristine sheet-type composite cathode acquired with our in-house XPS with an X-ray beam energy of 1486.6 eV. The BE was aligned using the adventitious carbon from the C 1s spectrum at 284.8 eV. The upper sketch illustrates the cathode surface that is exposed after coating of the sheet-type composite cathode onto the aluminum current collector (Al-CC), yielding the spectrum given by the green line. The lower sketch illustrates the cathode surface that is exposed in the *operando* cell, which is produced by the removal of the Al-CC (see Fig. 1 in the main text), with the corresponding spectrum given by the blue line.

3. *Operando* reference spectra for Ni 2p, C 1s, O 1s, and S 1s

3.1. Intensity normalization and BE-correction of the XPS data

To allow for a more quantitative analysis, all spectra shown in the main part were area normalized to the Ni 2p spectrum recorded at the respective SOC under OCV condition. All spectra are plotted in Fig. S7, separated into the first charge from BOT to 4.10 V_{Li} (left panel), the first discharge from 4.10 to 2.5 V_{Li} (middle panel), and the second charge from 2.50 to 5.50 V_{Li} (right panel), with the colors referring to the respective SOC. As can be seen, even though a different spot on the electrode was used for each measurement set at a given SOC (including a re-calibration of the focal point), the absolute counts per second are relatively constant. A summary of the integrated peak areas for the Ni 2p spectra is given in following Table S2. The minor differences are likely due to slight inhomogeneities in the coating and/or due to the geometry of the cell holder, where the incident beam was partially shielded towards one end of the slit. Nonetheless, owing to the minor variation of the absolute counts and the good signal-to-noise ratio, a normalization of the XPS spectra by the area of the Ni 2p spectra should be reliable.

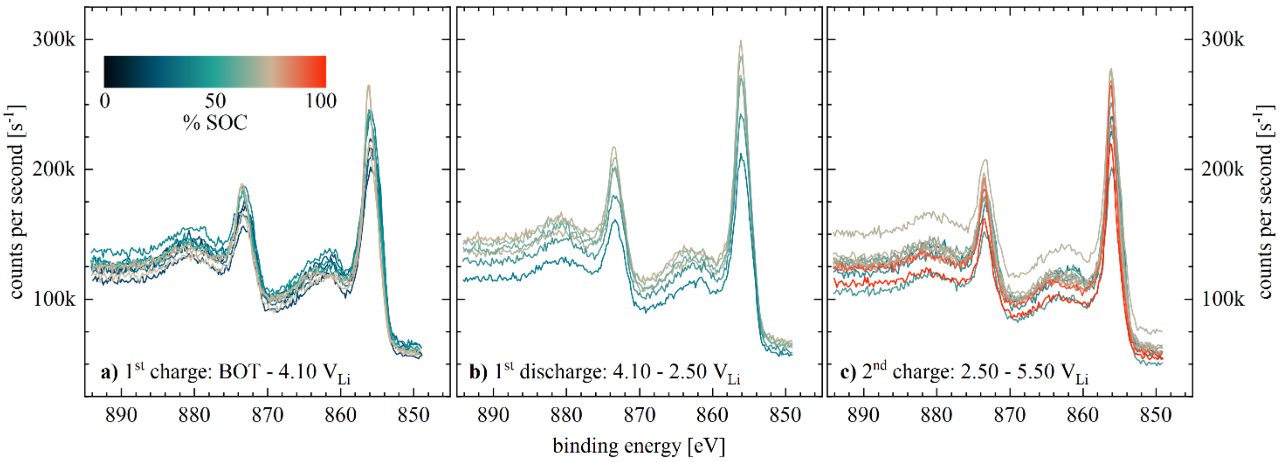

Figure S7: *Operando* Ni 2p spectra, recorded at the indicated SOCs over the course of the *operando* XPS measurements (for the associated potential versus time or SOC profiles see Fig. 3 in the main text): **a)** 1st charge from BOT to 4.10 V_{Li}; **b)** 1st discharge from 4.10 to 2.50 V_{Li}; **c)** 2nd charge from 2.50 to 5.50 V_{Li}. All binding energies were energy corrected to the layered oxide peak in the O 1s spectra, which was set to 529.3 eV.

To facilitate the analysis of the Ni 2p, and to highlight the reversibility of the redox, the Ni 2p spectra at BOT, 4.10 V_{Li} (69 % SOC), 2.50 V_{Li} (23 % SOC) and 5.50 V_{Li} (100 % SOC) are extracted from Figure S7, a Shirley background was subtracted, and the spectra were normalized to the maximum counts. The result is shown in Figure S8, where one can monitor the reversibility nicely throughout the spectra, especially visible at the Ni $2p_{3/2}$ peak and the satellites (i.e., 860-870 eV and 875-890 eV). As the Ni 2p region involves complex multiplet splitting and satellite features, requiring more in-depth analysis and theoretical modeling, we refrain from detailed Ni 2p oxidation state analysis during charge and discharge.

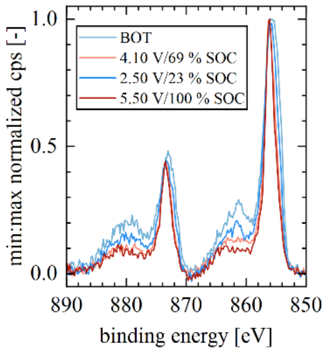

Figure S8: *Operando* Ni 2p spectra, recorded at the indicated SOCs over the course of the *operando* XPS measurements (for the associated potential versus time or SOC profiles see Fig. 3 in the main text), for BOT, 4.10 V_{Li} (69 % SOC), 2.50 V_{Li} (23 % SOC), and 5.50 V_{Li} (100 % SOC). All binding energies were energy corrected to the layered oxide peak in the O 1s spectra, which was set to 529.3 eV.

Table S2: Integrated areas of the Ni 2p core level as a function of SOC for the 1st charge (BOT - 4.10 V_{Li}), 1st discharge (4.10 - 2.50 V_{Li}) and the 2nd charge (2.50 - 5.50 V_{Li}).

**1st charge to U_{CV} = 4.10 V_{Li}**		**1st discharge to U_{CV} = 2.50 V_{Li}**		**2nd charge to U_{CV} = 5.50 V_{Li}**	
SOC [%]	Area [eV s^{-1}]	SOC [%]	Area [eV s^{-1}]	SOC [%]	Area [eV s^{-1}]
0	1206635.1	60	1113548.4	32	1133909.1
1	987871.5	52	1112569.0	40	1000189.9
10	1075861.7	43	1129055.0	49	867331.4
19	952882.0	34	1151200.1	57	1051522.9
28	1068799.1	26	1068013.3	66	827183.6
36	1113477.3	23	929108.0	74	890125.9
45	1044479.9			83	981443.5
54	955821.8			91	864496.0
62	1037020.8			95	839068.7
69	1033388.6			98	858511.5
				99	922527.8
				100	739098.1

The Ni-spectra themselves show changes at higher SOCs (reddish colored lines in the right panel of Fig. S7), where the Ni $2p_{3/2}$ peaks tilts towards higher BEs, which is indicative of an NiO-type surface layer formation.[9,10] Additionally, slight shifts of the Ni $2p_{3/2}$ features are also visible through charge and discharge. To analyze this, the Ni $2p_{3/2}$ BE after the energy correction to the O 1s MO_2 feature was extracted and is plotted as a function of SOC in Fig. S9. Here, a slight increase of the Ni $2p_{3/2}$ BE by

≈ 0.4 eV when increasing the SOC from 0 to 100 % can be observed, over which range the cathode potential increases from ≈ 3.0 - 5.5 V_{Li} (Fig. 3, main text). This trend is the opposite of what was observed in the literature, where the increase of the working electrode potential leads to a shift towards lower BEs, amounting to ≈ 1 eV/V.[11–13] The reason behind this is further elaborated in the main text and section 2 of the SI.

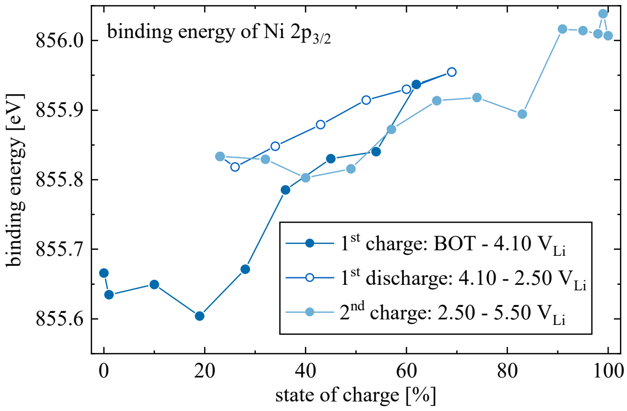

Figure S9: Binding energies of the Ni $2p_{3/2}$ *operando* spectra, recorded at the indicated SOCs (for the associated potential versus time or SOC profiles see Fig. 3, main text). All binding energies were energy corrected to the layered oxide in the O 1s spectra, which was set to 529.3 eV. The BE was extracted by fitting the Ni $2p_{3/2}$ peak by one GL(30) fit function. To convert the given SOCs into U_{CV}, please refer to Table S1.

Here, it is important to also analyze the C 1s spectra. Owing to the varying amount of adventitious carbon in combination with the different amounts of surface contaminants and binder, an energy correction via the adventitious carbon was not deemed reliable. Instead, the C 1s spectra were corrected to the BE of the O 1s MO_2 feature, as was done for all other core level spectra. The results are plotted in Fig. S10, where the typically used adventitious carbon BE of 284.8 eV is marked by the gray dashed lines.

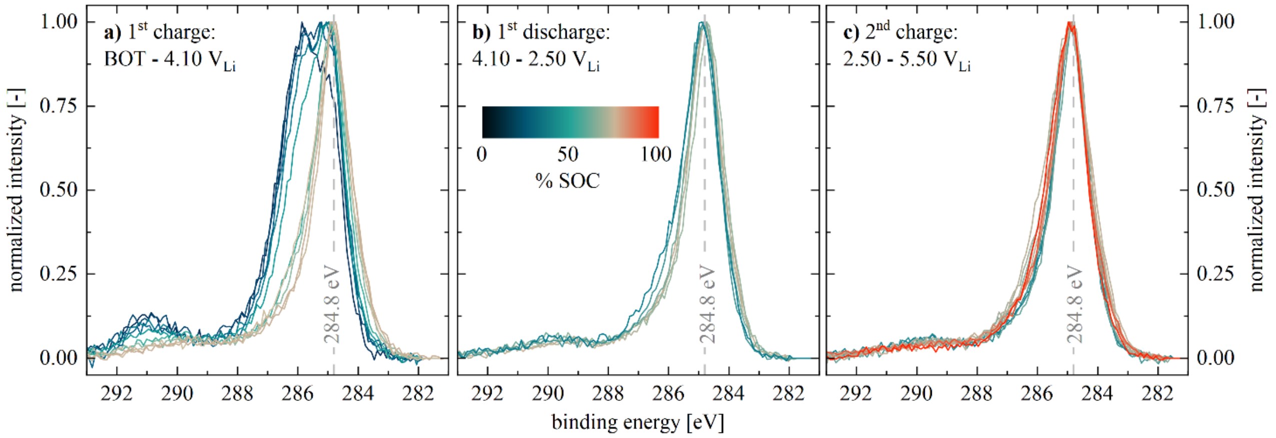

Figure S10: *Operando* C 1s spectra, recorded at the indicated SOCs (for the associated potential versus time or SOC profiles see Fig. 3 in the main text): **a)** 1st charge from BOT to 4.10 V_{Li}; **b)** 1st discharge from 4.10 to 2.50 V_{Li}; **c)** 2nd charge from 2.50 to 5.50 V_{Li}. All BEs were energy corrected to the layered oxide in the O 1s spectra (set to 529.3 eV). The typical adventitious carbon BE of 284.8 e is indicated by the dashed gray lines.

During the first charge, the peak at ≈ 291 eV, which is indicative for carbonates,[14,15] such as Li_2CO_3, substantially decreases at 36 % SOC, reached after a 0.5 h CV-hold at U_{CV} = 3.84 V_{Li} (left panel of Fig. S10); this potential coincides also with a decrease in oxygen-containing surface species, as inferred from the O 1s spectra (Fig. 4a; main text). Hence, this seems to mark the electrochemical stability limit of carbonates. This is accompanied by a significant change in the C 1s features between ≈ 284 - 288 eV: the initially broad peak near ≈ 286 eV with a shoulder at ≈ 284.8 eV changes into a rather narrow peak at ≈ 284.8 eV, indicating the loss of other C-O type surface contaminants (e.g., nickel carbonate basic hydrate).[16,17] While the peak at ≈ 284.8 eV corresponds to that of adventitious carbon, it likely also contains the signals from the C-H moieties of PIB. Since the C 1s spectra show two different species in the region where the signal for adventitious carbon is expected (lower panel of Fig. S5 and Fig. S10), the C 1s is not used for any assignment or analysis, and a more detailed discussion will be omitted.

Hence, as an alternative for the typically used adventitious carbon-based BE reference, one could consider using the sulfur spectra for referencing BEs, which show mostly well distinguishable features; however, since there is very little literature on the S 1s core level, this approach was not taken. Ni was also not considered as a BE reference, because Ni in the NCM material changes its redox state during charge and discharge and thus is not a reliable BE reference. For these reasons, the O 1s signal of the "MO_2" layered oxide from the NCM material at 529.3 eV was used instead for the binding energy alignment,[7,18] as this feature is strongly pronounced and clearly distinguishable throughout all SOCs. Hence, the O 1s spectra were fitted with two peaks to determine the MO_2 O1s peak energy, which was then shifted to the above-mentioned value of 529.3 eV. Throughout all SOCs, the thus applied BE shift of the raw data remained essentially constant, amounting to 3.90 ± 0.03 eV, which we ascribe to the work function of the analyzer. The essentially constant BE of the MO_2 feature can be observed in Fig. 3.4a, where the uncorrected BE of the MO_2 feature is plotted.

As discussed in the main text (see Fig. 4), the feature ascribed to the mixed oxygen-containing surface species ("RO_x") shifts towards lower BEs during the first charge, but stays constant during the first discharge and the second charge. Since the energy reference at each potential/SOC is performed with a nearly constant shift, this shift is not an artefact but can be observed both in the uncorrected (Fig. 3.4a), as well as in the BE-corrected plot in Fig. 3.4b.

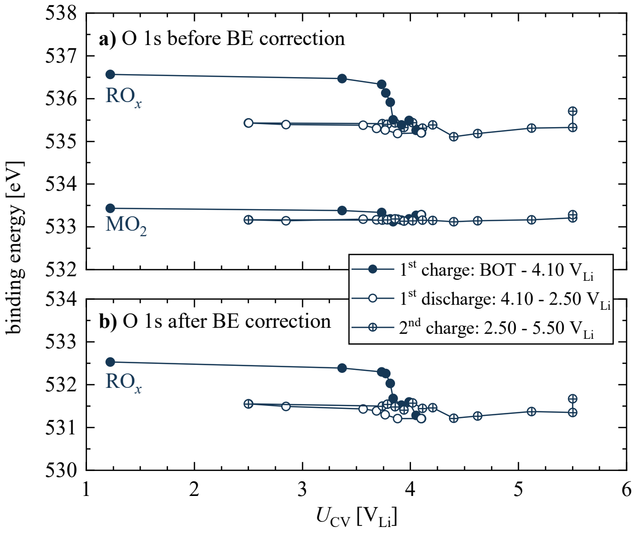

Figure S11: a) As-measured binding energies of the O 1s features assigned to the layered oxide "MO_2" and the oxygen-containing surface species "RO_x" during the first charge (BOT – 4.10 V_{Li}, filled circles), the first discharge (4.10 – 2.50 V_{Li}, hollow circles), and the second charge (2.50 – 5.50 V_{Li},) as a function of potential during the CV-hold (U_{CV}; see Table S1). **b)** BE of the "RO_x" feature when referenced to the BE of the "MO_2" feature set at 529.3 eV.

When analyzing the recorded binding energies for the electrolyte species, one can also observe that the individual species, as a function of potential, exhibit a non-uniform shift throughout the experiment. While these changes appear to be gradual, one, of course, needs to consider that this is an effect of the applied binding energy correction. This seems to be unlikely, as the observed changes occur in potential ranges where reactions involving the individual species would be expected. Hence, through the ongoing reactions, a change in the chemical composition of all species is expected, and therefore a slight shift of the BE seems plausible.

When analyzing the difference plots described in Fig. 4 (normalized by the respective Ni 2p peak area), it was noticed that during the first charge the change in the O 1s spectra of the RO_x feature exhibits an isosbestic point, which we ascribe to the conversion of species S to S^* (main text, discussion after Figs. 4 and 6). To quantify this conversion reaction, the absolute area of the difference plot in the spectral region of the RO_x feature was determined by integrating the difference plot from 530 - 538 eV and then dividing it by two, which will be referred to as A(Diff.)/2. Its absolute value is shown in Table S3, together with its values normalized by the area of the RO_x feature ($A(RO_x)$; determined with a single component fit), which provides three important insights for the first charge to $U_{CV} = 4.1$ V_{Li} and the first discharge to $U_{CV} = 2.5$ V_{Li}: i) the value of $A(RO_x)$ remains roughly constant (at a mean of ≈ 0.61, with a standard deviation of ≈ 5 %), indicating no net loss of oxygen species in the RO_x region; ii) the change occurs from BOT to ≈ 36 % SOC, after which A(Diff.)/2, referenced to $A(RO_x)$ stops increasing and plateaus out, remaining constant at ≈ 25 ± 3 % until the end of the first charge, indicating a full conversion; iii) the

percentage of species S that have transformed to S^* amounts to an average of 17 % during the first charge, meaning that only a relatively small fraction of surface species has transformed.

Table S3: Integrated areas of the RO_x ($A(RO_x)$), the area of the isosbestic point around 530 - 538 eV in the difference plot (A(Diff.)), divided by two, and the relative ratio between A(Diff.) and $A(RO_x)$ in percent, sorted by SOC for the 1st charge (BOT - 4.10 V_{Li}), 1st discharge (4.10 - 2.50 V_{Li}) and the 2nd charge (2.50 - 5.50 V_{Li}).

**1st charge to U_{CV} = 4.10 V_{Li}**				**1st discharge to U_{CV} = 2.50 V_{Li}**				**2nd charge to U_{CV} = 5.50 V_{Li}**			
SOC [%]	$A(RO_x)$ [-]	$\frac{A(Diff.)}{2}$ [-]	$\frac{\frac{A(Diff.)}{2}}{A(RO_x)}$ [%]	SOC [%]	$A(RO_x)$ [-]	$\frac{A(Diff.)}{2}$ [-]	$\frac{\frac{A(Diff.)}{2}}{A(RO_x)}$ [%]	SOC [%]	$A(RO_x)$ [-]	$\frac{A(Diff.)}{2}$ [-]	$\frac{\frac{A(Diff.)}{2}}{A(RO_x)}$ [%]
0	0.54	0	0	60	0.59	0.17	29	32	0.56	0.17	30
1	0.62	0.03	5	52	0.55	0.17	31	40	0.56	0.14	25
10	0.62	0.03	5	43	0.61	0.18	30	49	0.55	0.18	33
19	0.67	0.06	9	34	0.57	0.16	28	57	0.57	0.14	25
28	0.67	0.08	12	26	0.58	0.14	24	66	0.73	0.14	19
36	0.61	0.13	21	23	0.68	0.15	22	74	0.68	0.13	19
45	0.63	0.16	25					83	0.70	0.16	23
54	0.68	0.14	21					91	0.69	0.13	19
62	0.59	0.17	29					95	0.63	0.14	22
69	0.55	0.16	29					98	0.60	0.13	22
								99	0.55	0.13	24
								100	0.60	0.09	15

3.2. Evolution of the S 1s features and areas recorded for the operando cell

Figure S12 shows all S 1s spectra that were recorded in the *operando* cell at after each of the CV-holds during the first charge to $U_{CV} = 4.10$ V_{Li}, the first charge to $U_{CV} = 2.50$ V_{Li}, and the second charge to $U_{CV} = 5.50$ V_{Li}: the SOC values after each CV-hold step are given in the figure; the associated U_{CV} potentials can be found in Table S1.

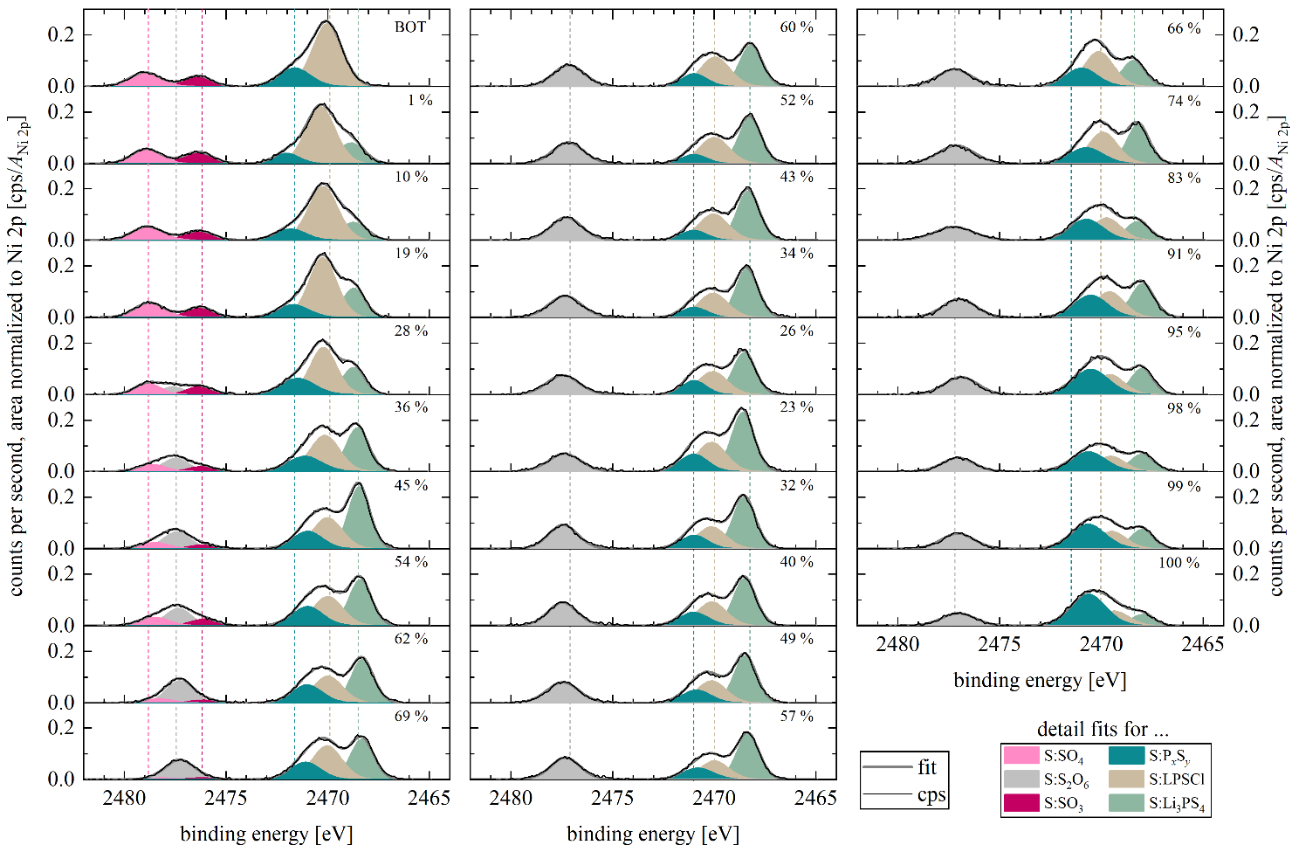

Figure S12: *Operando* XPS spectra of the S 1s region acquired at all the U_{CV} potentials applied to the *operando* cell (see Table S1) during: **a)** the first charge from BOT to $U_{CV} = 4.10$ V_{Li} (69 % SOC); **b)** the first discharge to $U_{CV} = 2.50$ V_{Li} (23 % SOC); and, **c)** the second charge to $U_{CV} = 5.50$ V_{Li} (100 % SOC). All spectra are normalized to the total Ni 2p peak area at the respective SOC and are BE-corrected to the O 1s feature from the lattice oxygen in the layered oxide structure of the NCM, which is fixed to be at 529.3 eV (see section 3.1 of the SI); furthermore, a Shirley background was subtracted from all spectra.

The above S 1s spectra can be evaluated more quantitatively by considering that the kinetic energy of the photoelectrons of all the sulfur species (BEs ranging between ≈ 2466 - 2480 eV), recorded at a beam energy of 4000 eV, is very similar (E_{kin} ≈ 1520 - 1534 eV), so that the analyzer sensitivity and the mean free path of the associated S 1s photoelectrons ($\lambda_{S\ 1s}$) are essentially identical. In this case, the relative amount of the different sulfur species can be determined simply by their relative peak areas. The evolution of the areas of the S:SO_4, the S:SO_3, and the S:S_2O_6 peaks over the course of the first charge to $U_{CV} = 4.10$ V_{Li} (normalized by the Ni 2p area and determined from Fig. S12a) is shown in Fig. S13a, together with the summed up area of these three species. This indicates that the S:S_2O_6 species begin to

form between U_{CV} = 3.77 - 3.81 V_{Li} (i.e., between 19 - 28 % SOC), accompanied by the roughly equimolar consumption of the S:SO_4 and S:SO_3 species. Furthermore, the sum of the areas of these species remains essentially constant, which means that no elemental sulfur is being formed by the reaction, as sulfur would sublime into vacuum. As a matter of fact, after their initial formation, the area of the S:S_2O_6 species remains essentially constant until a potential of U_{CV} = 4.62 V_{Li} (95 % SOC) in the second charge (solid black symbols in Fig. 3.6c), beyond which the area decreases, indicating the oxidative decomposition of the S:S_2O_6 species.

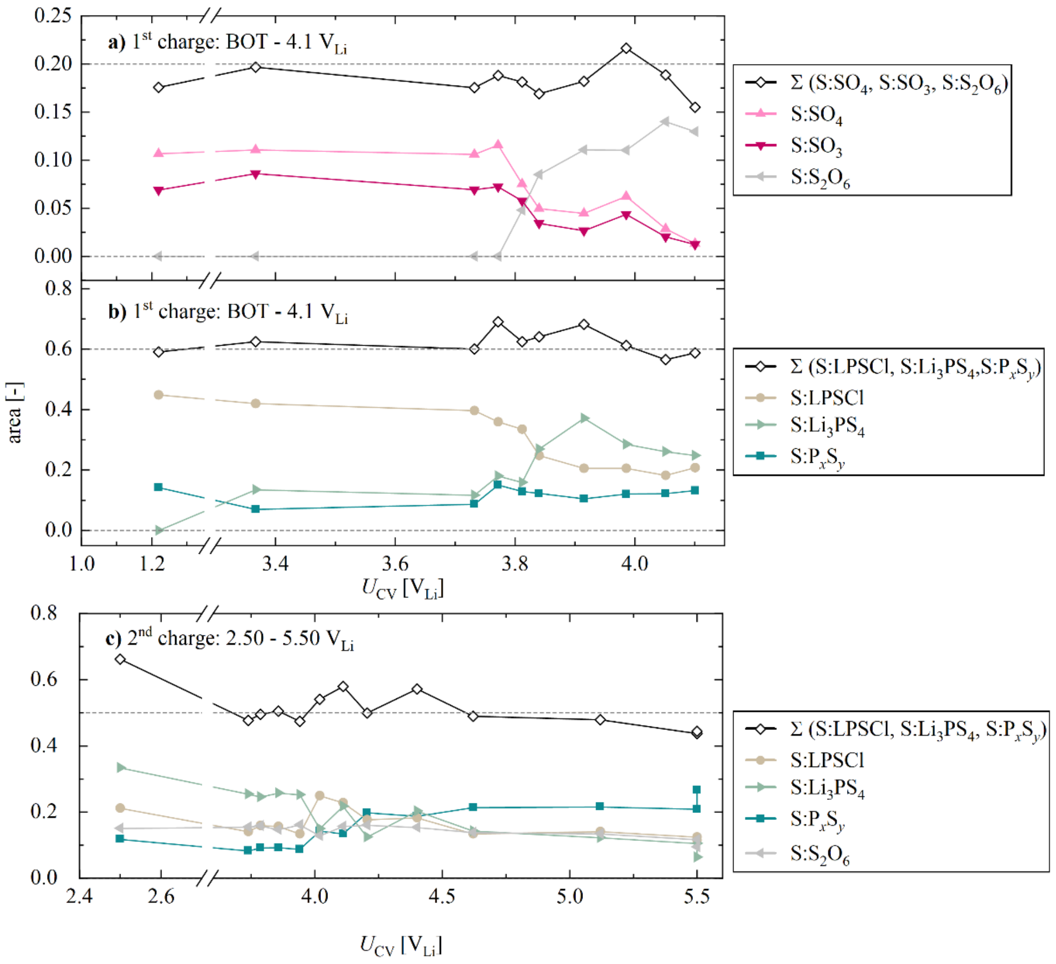

Figure S13: Areas of the individual species from the S 1s core level peaks (normalized to the area of the Ni 2p peak), determined from the data in Fig. S12 by using a GL(30) fit function after a Shirley background subtraction. **a)** Evolution of the areas of the S:SO_4, S:SO_3, and S:S_2O_6 features (solid symbols) over the course of the first charge to U_{CV} = 4.1 V_{Li} (corresponding to 69 % SOC; see Table S1); in addition, the sum of the areas of these three species is given (open symbols). **b)** Evolution of the areas of the S:LPSCl, S:Li_3PS_4, and S:P_xS_y features (solid symbols) over the course of the first charge to U_{CV} = 4.1 V_{Li}.); in addition, the sum of the areas of these three species is given (open symbols). **c)** Evolution of the areas of the S:LPSCl, S:Li_3PS_4, and S:P_xS_y features as well as of the S:S_2O_6 feature over the course of the second charge to U_{CV} = 5.50 V_{Li} (100 % SOC); in addition, the sum of the areas of the S:LPSCl, S:Li_3PS_4, and S:P_xS_y features is given (open symbols).

Fig. S13b provides a similar analysis for the S 1s peaks in the BE range of 2466 - 2474 eV over the course of the first charge to, showing the peak areas for the S:LPSCl (beige), the S:Li_3PS_4 (light green), and the

S:P_xS_y species (dark green), as well as of the sum of the area of these species. Quite clearly, between U_{CV} = 3.73 - 3.77 V_{Li} (10 - 19 % SOC), the relative amounts of the S:LPSCl species decreases while that of the S:L_3PS_4 species increases; however, the sum of the S 1s area in this BE region remains constant, indicating that there is no net loss of sulfur or, in other words, that no elemental sulfur is formed. During the second charge, shown in Fig. S13c, the amount of the S:P_xS_y species increases at $U_{CV} > 4.1$ V_{Li}, concomitant with a decrease of that of the S:L_3PS_4 species. At the same time, the total amount of sulfur decreases, suggesting sublimation and loss of elemental sulfur. Here we should note that the Ni 2p-normalized area of all the species plotted in Fig. S13c at U_{CV} = 2.5 V_{Li} (i.e., left-most data points) appear too large in comparison to the other values. This might originate from an artefact of the Ni 2p measurement, which for this measurement point yielded an absolute area of that was ≈ 13/18 % smaller than that obtained for directly preceding/following measurement points . If one were to use the Ni 2p area value of the following measurement point for area normalization, the total area would equate to 0.54 and would then be quite consistent with preceding/following points.

To further facilitate the analysis of the S 1s spectra in *operando*, the binding energy was extracted from the GL(30) fits shown in Fig. S12 and is plotted as a function of SOC in Fig. S14. Here, one can see how over the course of cycling, only gradual changes of the BE for the various S-containing species are visible.

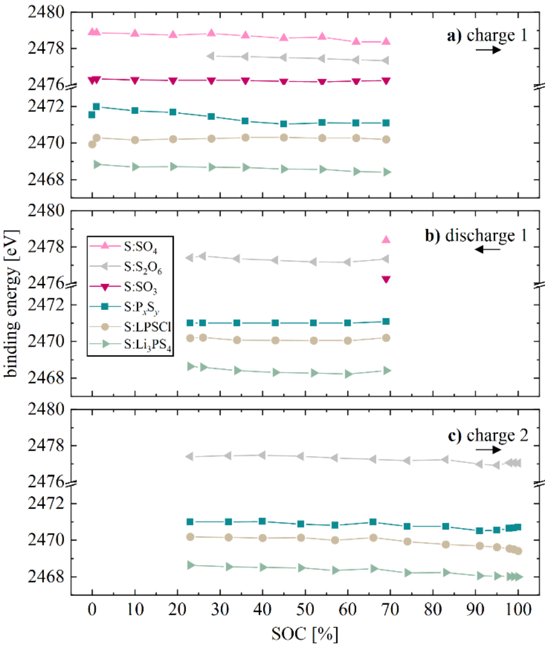

Figure S14: BEs of the individual species from the S 1s core level peaks, determined from the data in Fig. S11 by using a GL(30) fit function after a Shirley background subtraction. **a)** Evolution of the BEs of the S:LPSCl, S:Li_3PS_4, and S:P_xS_y features as well as of the S:SO_4, S:SO_3 and S:S_2O_6 feature over the course of the first charge to U_{CV} = 4.1 V_{Li} (corresponding to 69 % SOC; see Table S1) and **b)** evolution of the BEs over the course of the first discharge to U_{CV} = 2.5 V_{Li} (23 % SOC). **c)** Evolution of the BEs of the S:LPSCl, S:Li_3PS_4, and S:P_xS_y features as well as of the S:S_2O_6 feature over the course of the second charge to U_{CV} = 5.50 V_{Li} (100 % SOC).

3.3. Correlation between the BE of the SE related signals and the WE potential

Lastly, the absence of the shift of the BEs with the applied electrochemical potential needs to be discussed. In principle, the WE was grounded to the analyzer, and all electrically conductive components should not show a shift, while all electrically insulated components should. However, an inherent potential-dependent shift was observed. While this could either mean that all components have a high conductivity, which is unlikely, because the cell did neither short nor self-discharge, several other hypotheses need to be considered:

- Accidentally "overcorrecting" the BE based on the MO_2 feature can be excluded; as the uncorrected raw data show (Figure S11a) that this would simply have led to a slight shift during the first charge, and for the rest of the experiment a constant offset is observed, regardless of charge and/or discharge.
- Other literature reports have shown that upon oxidation of the SE at the interface to the CAM, transition metal sulfides form, which have been found to be electronically conductive.[58,67] While our experiments do not allow us to confirm/exclude this possibility, if metal sulfides were indeed formed, the layer or CEI produced by the oxidative LPSCl decomposition would become a mixed ionic/electronic (semi)conductor, in which case no potential-induced BE shift of the species contained in the CEI would be expected.
- The CAM itself has a reasonably good electronic conductivity, as it is Ni-rich, so that it should be unaffected by the applied potential, which indeed appears to be the case, as the BE of the MO_2 feature that is part of the CAM does not shift with the applied potential (Figure S11a).
- The individual species shift slightly in BE throughout cycling. While we interpret this as range of phase of the continuously oxidizing and degrading species, this slight shift needs to be considered as the error range for fitting and hence for the determined BEs, amounting to $\pm$ 0.2 eV over the entire SOC range. We hope that future studies can bring more clarity into these shifts.
- Lastly, as evidenced by both the electrochemical measurements in the *operando* cell (Fig. 3, main text) and the CV experiments (Fig. S2), continuous oxidation of the SE over the course of the *operando* experiment is expected. Furthermore, the SE oxidation is triggered by the potential (rather than by the applied current), the SE degradation will continue in the OCV periods during which the XPS spectra are recorded, the net current is not zero, but governed by the SE degradation.

A more detailed analysis is beyond the scope of this study but will hopefully soon be elucidated.

4. XPS measurements of reference samples

4.1. S 1s spectra of reference samples acquired at the ALS

Prior to the *operando* XPS measurements, the XPS spectra of a pristine sheet-type composite cathode and a pristine sheet-type separator sheet (experimental section of the main text) were acquired at a beam energy of 4 keV. For this, both cathode and separator were glued to the front plate of the cell case and the spectra were recorded prior to the electrochemical cycling (i.e., at BOT).

The XPS spectra were acquired using the identical procedure as described above for the *operando* cell measurements. To reference the BE, first the sheet-type cathode was measured, whereby the BE was referenced to the layered oxide at 529.3 eV, as done before. Due to the absence of layered oxide in the sheet-type separator, the BE of LPSCl in the Cl 1s spectrum of the sheet-type cathode was determined and used to reference the spectra of the sheet-type separator. Based on the literature, the assignments of the various S 1s features observed for the sheet-type cathode (Fig. S15a) and the sheet-type separator (Fig. S15b) are: 1 = sulfates at ≈ 2479.0 eV, 2 = sulfites at ≈ 2476.4 eV, 3 = polysulfides at ≈ 2471.6 eV, and 4 = LPSCl at ≈ 2470.0 eV. The sheet-type separator only shows the single S 1s feature that is expected for a pure LPSCl material (peak 4 in Fig. S15b). On the other hand, even though the sheet-type cathode was prepared by the analogous slurry-based preparation method, using the same LPSCl SE and the same PIB binder as for the sheet-type separator, additional XPS features that can be ascribed to sulfates (feature 1), sulfites (feature 2), and polysulfides (feature 3) can be observed.

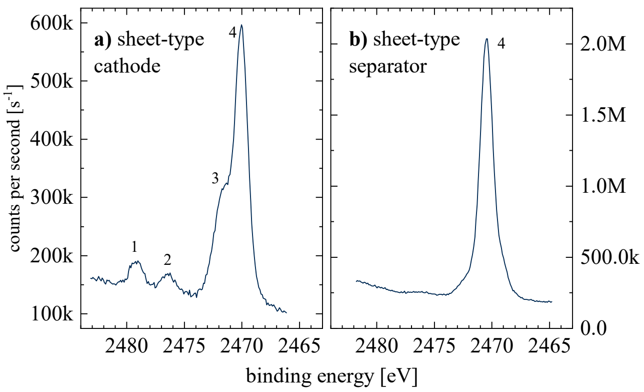

Figure S15: Reference sample XPS measurements of the S 1s region at the ALS with an X-ray beam energy of 4 keV: **a)** pristine sheet-type composite cathode, acquired from the as-coated side of the cathode (i.e., the side opposite of the aluminum current collector foil); **b)** pristine sheet-type separator. Based on the literature (see above), the clearly visible spectral features marked by the numerical labels in the figure can be ascribed to: 1 = sulfates at ≈ 2479.0 eV, 2 = sulfites at ≈ 2476.4 eV, 3 = polysulfides at ≈ 2471.6 eV, and 4 = LPSCl at ≈ 2470.0 eV.

4.2. S 2p spectra of reference samples acquired with an in-house XPS

To understand the origin of these differences, the S 2p spectra of a sheet-type cathode, a sheet-type separator, and of the pristine NCM powder were reexamined, since contrary to the S 1s spectra, numerous

literature references exist for the assignment of the S 2p spectra. These S 2p spectra were recorded with our in-house XPS equipped with an Al K_α source (beam energy of 1486 eV) and are shown in Fig. S16. For the sheet-type cathode (panel a) and separator (panel b), LPSCl can be identified at ≈ 161 eV (here referred to as feature 3), with a FWHM of 1.1 eV.[19,20] This FWHM is in accordance with literature, and hence we can conclude that the binder in the sheet-type electrode, in comparison to powder cells, does not add beak broadening due to its insulating nature, and spectra are comparable to studies with powder-based cells.[3] Interestingly, sulfates/sulfites (features 1/2, at BE ≈ 169 eV) are again not present in the sheet-type separator, but can be identified in the NCM-containing samples, namely for the sheet-type cathode (panel a) and for the NCM powder (panel c). Hence, the origin of the sulfates in the S 1s *operando* spectra can be linked to the NCM material, likely originating from its synthesis.[21,22]

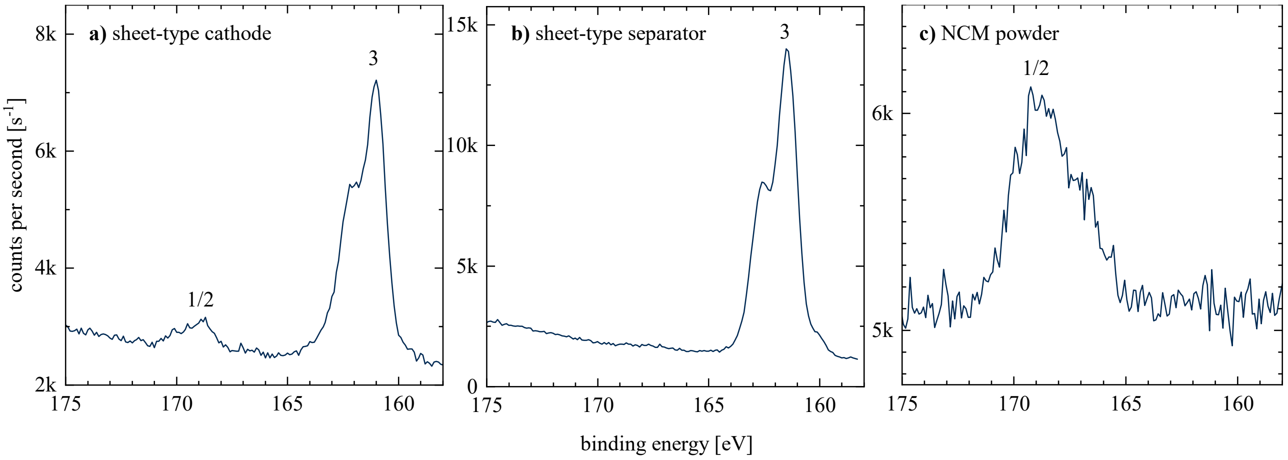

Figure S16: Reference sample measurements of the S 2p region with an Al K_α source at 1486 eV beam energy for: **a)** a pristine composite cathode; **b)** a pristine sheet-type separator; and, **c)** a pristine NCM powder. The clearly visible features can be assigned to sulfites/sulfates (labelled as 1/2) and to pristine LPSCl (labelled as 3).

4.3. S 1s spectra of reference samples acquired with different instruments and literature comparison

To further confirm the nature of the species, ammonia sulfate, sodium dithionate, and sodium sulfite were measured using the Ag L_α source at our in-house XPS in order to determine the associated S 1s BEs and to compare them to the values measured at the ALS as well as those reported in the literature.

For that, $(NH_4)_2SO_4$ (Sigma Aldrich, USA) and Na_2SO_3 (Sigma Aldrich, USA) were used as-received, and $Na_6S_2O_6$ was synthesized according to Schindler et al.[23] The powders were then mounted onto a stainless-steel holder and transferred into the XPS without exposure to air. Spectra of the samples were then recorded between 2490 - 2455 eV with a step size of 0.2 eV and a dwell time of 4 s, using an emission current of 20 mA. The extracted BEs for the various species are referred to as "in-house" and are summarized in Tab. S4, together with the BEs for the assigned species from the *operando* measurements

(referred to as “ALS”) as well as with BE values found in the literature. As can be seen from Tab. S4, the measured BEs for the various species are consistent with the assignments that have been made in the main text. For Na_2SO_3, two species were found in the measured reference; here, the species labeled with * is considered to be the sulfite.

Table S4: Binding energies and full-width-at-half-maximum values (FWHM, in brackets) for S 1s features of reference compounds measured at our in-house XPS using an Ag L_α-source (2699 eV), at the ALS (at 4000 eV), and BE values reported in the literature.

		S 1s BE (FWHM) [eV]
$(NH_4)_2SO_4$	In-house	2478.0 (1.9)
	ALS	2479.0 (1.8), meas. at BOT
	Literature	2478[13]<br>2479.2[24]
$Na_2S_2O_6$	In-house	2477.2 (1.8)
	ALS	2477.3 (1.7), meas. at U_{CV} = 4.10 V_{Li}
	Literature	2477[13]
Na_2SO_3	In-house	2475.2 (1.6)<br>2477.6 (1.8)*
	ALS	2476.4 (1.5), meas. at BOT
	Literature	2476[13]<br>2476.7[24]

References

(1) Santhosha, A. L.; Medenbach, L.; Buchheim, J. R.; Adelhelm, P. The Indium−Lithium Electrode in Solid-State Lithium-Ion Batteries: Phase Formation, Redox Potentials, and Interface Stability. *Batter. Supercaps* **2019**, *2* (6), 524–529. https://doi.org/10.1002/batt.201800149.

(2) Sedlmeier, C.; Schuster, R.; Schramm, C.; Gasteiger, H. A. A Micro-Reference Electrode for Electrode-Resolved Impedance and Potential Measurements in All-Solid-State Battery Pouch Cells and Its Application to the Study of Indium-Lithium Anodes. *J. Electrochem. Soc.* **2023**, *170* (3), 030536. https://doi.org/10.1149/1945-7111/acc699.

(3) Sedlmeier, C.; Kutsch, T.; Schuster, R.; Hartmann, L.; Bublitz, R.; Tominac, M.; Bohn, M.; Gasteiger, H. A. From Powder to Sheets: A Comparative Electrolyte Study for Slurry-Based Processed Solid Electrolyte/Binder-Sheets as Separators in All-Solid-State Batteries. *J. Electrochem. Soc.* **2022**, *169* (7), 070508. https://doi.org/10.1149/1945-7111/ac7e76.

(4) Tan, D. H. S.; Wu, E. A.; Nguyen, H.; Chen, Z.; Marple, M. A. T.; Doux, J.-M.; Wang, X.; Yang, H.; Banerjee, A.; Meng, Y. S. Elucidating Reversible Electrochemical Redox of Li 6 PS 5 Cl Solid Electrolyte. *ACS Energy Lett.* **2019**, *4* (10), 2418–2427. https://doi.org/10.1021/acsenergylett.9b01693.

(5) Oswald, S.; Gasteiger, H. A. The Structural Stability Limit of Layered Lithium Transition Metal Oxides Due to Oxygen Release at High State of Charge and Its Dependence on the Nickel Content. *J. Electrochem. Soc.* **2023**, *170* (3), 030506. https://doi.org/10.1149/1945-7111/acbf80.

(6) Tanuma, S.; Powell, C. J.; Penn, D. R. Calculations of Electron Inelastic Mean Free Paths. V. Data for 14 Organic Compounds over the 50–2000 EV Range. *Surf. Interface Anal.* **1994**, *21* (3), 165–176. https://doi.org/10.1002/sia.740210302.

(7) Freiberg, A. T. S.; Qian, S.; Wandt, J.; Gasteiger, H. A.; Crumlin, E. J. Surface Oxygen Depletion of Layered Transition Metal Oxides in Li-Ion Batteries Studied by Operando Ambient Pressure X-Ray Photoelectron Spectroscopy. *ACS Appl. Mater. Interfaces* **2023**, *15* (3), 4743–4754. https://doi.org/10.1021/acsami.2c19008.

(8) Biesinger, M. C.; Payne, B. P.; Grosvenor, A. P.; Lau, L. W. M.; Gerson, A. R.; Smart, R. S. C. Resolving Surface Chemical States in XPS Analysis of First Row Transition Metals, Oxides and Hydroxides: Cr, Mn, Fe, Co and Ni. *Appl. Surf. Sci.* **2011**, *257* (7), 2717–2730. https://doi.org/10.1016/j.apsusc.2010.10.051.

(9) Mansour, A. N. Characterization of LiNiO2 by XPS. *Surf. Sci. Spectra* **1994**, *3* (3), 279–286.

https://doi.org/10.1116/1.1247757.

(10) Mansour, A. N. Characterization of NiO by XPS. *Surf. Sci. Spectra* **1994**, *3* (3), 231–238. https://doi.org/10.1116/1.1247751.

(11) Wu, X.; Villevieille, C.; Novák, P.; El Kazzi, M. Monitoring the Chemical and Electronic Properties of Electrolyte-Electrode Interfaces in All-Solid-State Batteries Using: Operando X-Ray Photoelectron Spectroscopy. *Phys. Chem. Chem. Phys.* **2018**, *20* (16), 11123–11129. https://doi.org/10.1039/c8cp01213j.

(12) Yu, L.; Takagi, Y.; Nakamura, T.; Sekizawa, O.; Sakata, T.; Uruga, T.; Tada, M.; Iwasawa, Y.; Samjeské, G.; Yokoyama, T. Non-Contact Electric Potential Measurements of Electrode Components in an Operating Polymer Electrolyte Fuel Cell by near Ambient Pressure XPS. *Phys. Chem. Chem. Phys.* **2017**, *19* (45), 30798–30803. https://doi.org/10.1039/c7cp05436j.

(13) Yu, L.; Takagi, Y.; Nakamura, T.; Sakata, T.; Uruga, T.; Tada, M.; Iwasawa, Y.; Masaoka, S.; Yokoyama, T. Operando Observation of Sulfur Species Poisoning Polymer Electrolyte Fuel Cell Studied by Near Ambient Pressure Hard X-Ray Photoelectron Spectroscopy. *J. Phys. Chem. C* **2019**, *123* (1), 603–611. https://doi.org/10.1021/acs.jpcc.8b10611.

(14) Kozen, A. C.; Pearse, A. J.; Lin, C.-F.; Schroeder, M. A.; Noked, M.; Lee, S. B.; Rubloff, G. W. Atomic Layer Deposition and in Situ Characterization of Ultraclean Lithium Oxide and Lithium Hydroxide. *J. Phys. Chem. C* **2014**, *118* (48), 27749–27753. https://doi.org/10.1021/jp509298r.

(15) Etxebarria, A.; Yun, D.-J.; Blum, M.; Ye, Y.; Sun, M.; Lee, K.-J.; Su, H.; Muñoz-Márquez, M. Á.; Ross, P. N.; Crumlin, E. J. Revealing In Situ Li Metal Anode Surface Evolution upon Exposure to CO 2 Using Ambient Pressure X-Ray Photoelectron Spectroscopy. *ACS Appl. Mater. Interfaces* **2020**, *12* (23), 26607–26613. https://doi.org/10.1021/acsami.0c04282.

(16) Bhojane, P.; Sinha, L.; Goutam, U. K.; Shirage, P. M. A 3D Mesoporous Flowers of Nickel Carbonate Hydroxide Hydrate for High-Performance Electrochemical Energy Storage Application. *Electrochim. Acta* **2019**, *296*, 112–119. https://doi.org/10.1016/j.electacta.2018.11.025.

(17) Jung, R.; Morasch, R.; Karayaylali, P.; Phillips, K.; Maglia, F.; Stinner, C.; Shao-Horn, Y.; Gasteiger, H. A. Effect of Ambient Storage on the Degradation of Ni-Rich Positive Electrode Materials (NMC811) for Li-Ion Batteries. *J. Electrochem. Soc.* **2018**, *165* (2), A132–A141. https://doi.org/10.1149/2.0401802jes.

(18) Friedrich, F.; Strehle, B.; Freiberg, A. T. S.; Kleiner, K.; Day, S. J.; Erk, C.; Piana, M.; Gasteiger, H. A. Editors' Choice—Capacity Fading Mechanisms of NCM-811 Cathodes in Lithium-Ion Batteries Studied by X-Ray Diffraction and Other Diagnostics. *J. Electrochem. Soc.* **2019**, *166* (15),

A3760–A3774. https://doi.org/10.1149/2.0821915jes.

(19) Koerver, R.; Walther, F.; Aygün, I.; Sann, J.; Dietrich, C.; Zeier, W. G.; Janek, J. Redox-Active Cathode Interphases in Solid-State Batteries. *J. Mater. Chem. A* **2017**, *5* (43), 22750–22760. https://doi.org/10.1039/C7TA07641J.

(20) Auvergniot, J.; Cassel, A.; Ledeuil, J. B.; Viallet, V.; Seznec, V.; Dedryvère, R. Interface Stability of Argyrodite Li6PS5Cl toward LiCoO2, LiNi1/3Co1/3Mn1/3O2, and LiMn2O4 in Bulk All-Solid-State Batteries. *Chem. Mater.* **2017**, *29* (9), 3883–3890. https://doi.org/10.1021/acs.chemmater.6b04990.

(21) Oswald, S.; Wilhelm, R.; Kratky, T.; Szentmiklósi, L.; Maróti, B.; Harsányi, I.; Hallweger, S. A.; Kieslich, G.; Günther, S.; Gasteiger, H. A. Effect of Washing on Nickel-Rich NCMs: Methodology to Quantify the Lithium/Proton-Exchange Kinetics, the Inserted Protons, and the Structural Changes. *J. Mater. Chem. A* **2024**, *12* (37), 25140–25164. https://doi.org/10.1039/D4TA03319A.

(22) Hartmann, L.; Ching, C. H.; Kipfer, T.; Koch, M.; Gasteiger, H. A. Surface-Stabilization of LMR-NCM by Washing with Aqueous Buffers to Reduce Gassing and Improve Cycle-Life. *J. Electrochem. Soc.* **2022**, *169* (7), 070516. https://doi.org/10.1149/1945-7111/ac7ef0.

(23) Schindler, M.; Couvrat, N.; Cartigny, Y.; Brandel, C.; Coquerel, G. Synthesis and Characterization of Sodium Dithionate and Its Dihydrate. *Chem. Eng. Technol.* **2019**, *42* (7), 1446–1451. https://doi.org/10.1002/ceat.201800705.

(24) Aktekin, B.; Kataev, E.; Riegger, L. M.; Garcia-Diez, R.; Chalkley, Z.; Becker, J.; Wilks, R. G.; Henss, A.; Bär, M.; Janek, J. Operando Photoelectron Spectroscopy Analysis of Li6PS5Cl Electrochemical Decomposition Reactions in Solid-State Batteries. *ACS Energy Lett.* **2024**, *9* (7), 3492–3500. https://doi.org/10.1021/acsenergylett.4c01072.
